# Machine Learning Approaches in High Myopia: Systematic Review and Meta-Analysis

**DOI:** 10.2196/57644

**Published:** 2025-01-03

**Authors:** Huiyi Zuo, Baoyu Huang, Jian He, Liying Fang, Minli Huang

**Affiliations:** 1 Ophthalmology Department First Affiliated Hospital of GuangXi Medical University Nanning China

**Keywords:** high myopia, pathological myopia, high myopia-associated glaucoma, machine learning, deep learning

## Abstract

**Background:**

In recent years, with the rapid development of machine learning (ML), it has gained widespread attention from researchers in clinical practice. ML models appear to demonstrate promising accuracy in the diagnosis of complex diseases, as well as in predicting disease progression and prognosis. Some studies have applied it to ophthalmology, primarily for the diagnosis of pathologic myopia and high myopia-associated glaucoma, as well as for predicting the progression of high myopia. ML-based detection still requires evidence-based validation to prove its accuracy and feasibility.

**Objective:**

This study aims to discern the performance of ML methods in detecting high myopia and pathologic myopia in clinical practice, thereby providing evidence-based support for the future development and refinement of intelligent diagnostic or predictive tools.

**Methods:**

PubMed, Cochrane, Embase, and Web of Science were thoroughly retrieved up to September 3, 2023. The prediction model risk of bias assessment tool was leveraged to appraise the risk of bias in the eligible studies. The meta-analysis was implemented using a bivariate mixed-effects model. In the validation set, subgroup analyses were conducted based on the ML target events (diagnosis and prediction of high myopia and diagnosis of pathological myopia and high myopia-associated glaucoma) and modeling methods.

**Results:**

This study ultimately included 45 studies, of which 32 were used for quantitative meta-analysis. The meta-analysis results unveiled that for the diagnosis of pathologic myopia, the summary receiver operating characteristic (SROC), sensitivity, and specificity of ML were 0.97 (95% CI 0.95-0.98), 0.91 (95% CI 0.89-0.92), and 0.95 (95% CI 0.94-0.97), respectively. Specifically, deep learning (DL) showed an SROC of 0.97 (95% CI 0.95-0.98), sensitivity of 0.92 (95% CI 0.90-0.93), and specificity of 0.96 (95% CI 0.95-0.97), while conventional ML (non-DL) showed an SROC of 0.86 (95% CI 0.75-0.92), sensitivity of 0.77 (95% CI 0.69-0.84), and specificity of 0.85 (95% CI 0.75-0.92). For the diagnosis and prediction of high myopia, the SROC, sensitivity, and specificity of ML were 0.98 (95% CI 0.96-0.99), 0.94 (95% CI 0.90-0.96), and 0.94 (95% CI 0.88-0.97), respectively. For the diagnosis of high myopia-associated glaucoma, the SROC, sensitivity, and specificity of ML were 0.96 (95% CI 0.94-0.97), 0.92 (95% CI 0.85-0.96), and 0.88 (95% CI 0.67-0.96), respectively.

**Conclusions:**

ML demonstrated highly promising accuracy in diagnosing high myopia and pathologic myopia. Moreover, based on the limited evidence available, we also found that ML appeared to have favorable accuracy in predicting the risk of developing high myopia in the future. DL can be used as a potential method for intelligent image processing and intelligent recognition, and intelligent examination tools can be developed in subsequent research to provide help for areas where medical resources are scarce.

**Trial Registration:**

PROSPERO CRD42023470820; https://tinyurl.com/2xexp738

## Introduction

Myopia is currently widely regarded as a significant public health issue, leading to substantial vision loss and serving as a risk factor for a range of other serious ocular diseases. It is estimated that by 2050, 4.758 billion people (49.8% of the world population) and 938 million people (9.8% of the world population) will suffer from myopia and high myopia, respectively [1]. A recent meta-analysis study proposed that the global economic burden due to productivity losses from uncorrected myopia and myopic macular degeneration is estimated to reach US $250 billion [2]. Therefore, the prevention of high myopia as well as the diagnosis and treatment of pathological myopia remain a formidable societal challenge.

High myopia is defined as the spherical equivalent ≤–6.0 diopter [3] when the accommodation of the eye is relaxed. However, increased severity of myopia and elongation of the eye’s axial length could alter the posterior segment structures, causing posterior scleral staphyloma, myopic macular degeneration, and optic neuropathy related to high myopia, potentially leading to the loss of best-corrected visual acuity [3]. High myopia-related fundus lesions stand as an important contributing factor to blindness across the world as well as in China [4]. The detection of high myopia hinges primarily on artificial auxiliary techniques, like refraction detection, fundus examination, measurement of axial length, and fundus photography. Nevertheless, manual examination and analysis by ophthalmologists are still essential, necessitating a significant investment of time and effort [5]. Additionally, in regions with limited medical resources, the shortage of ophthalmologists and medical equipment impedes the early and accurate identification of high-risk patients with high myopia, resulting in missed opportunities for optimal treatment. Therefore, forecasting the risk of high myopia and precisely diagnosing pathological myopia are currently major research focus.

With the rapid advances in computing technology and the ongoing refinement of statistical theory, machine learning (ML) has gradually been promoted and applied in clinical practice. For instance, ML can not only improve image quality, reduce misregistration, and simulate attenuation correction imaging in core cardiology [6], but also be used for cancer screening (detection of lesions), characterization and grading of tumors, and prognosis prediction, thus facilitating clinical decision-making [7]. Since fundus images are noncontact, noninvasive, low-cost, easily accessible, and easy to process, ML has been extensively used to diagnose common retinal diseases, including diabetic retinopathy [8-10], macular degeneration [10], and glaucoma [11-13]. ML has been applied to various image-processing tasks. Novel techniques for analyzing fundus images of high myopia and pathological myopia are continuously emerging [14,15]. However, the accuracy of these ML detections has not been systematically studied. Consequently, the present study was executed to comprehensively describe the accuracy of ML in detecting different degrees of lesions in high myopia, furnishing an evidence-based reference for subsequent lesion management.

## Methods

### Study Registration

This study was implemented as per the PRISMA (Preferred Reporting Items for Systematic Reviews and Meta-Analyses) 2020 guidelines and prospectively registered with PROSPERO (ID: CRD42023470820). The PRISMA checklist is available in Multimedia Appendix 1.

### Inclusion and Exclusion Criteria

We established detailed inclusion and exclusion criteria for this systematic review. To enhance visualization, these criteria are presented in tabular form (Textbox 1).

**Inclusion and exclusion criteria.**

**Inclusion criteria**

- Study type: (1) case-control, cohort, nested case-control, and case-cohort studies and (2) studies reported in English.
- Machine learning (ML): studies that fully constructed ML models for the prediction or diagnosis of high myopia, the diagnosis of pathological myopia, or the diagnosis of high myopia-associated glaucoma.
- Outcome measures: at least one of the following outcome indicators were reported: receiver operating characteristic (ROC), *c*-index, sensitivity, specificity, accuracy, recovery rate, accuracy rate, confusion matrix, *F*_1_-score, and calibration curve.
- Datasets: (1) some studies lacked independent validation sets, and only *k*-fold cross-validation was leveraged to verify the effect of the constructed mode; and (2) in some publicly available datasets, particularly those involving medical imaging, different studies have reported the efficiency of varying ML methods.

**Exclusion criteria**

- Study type: (1) meta, review, guide, expert opinion; and (2) studies with too few samples (less than 20 cases).
- ML: literature that only executed the risk factor analysis but did not develop a complete ML mode.
- Outcome measures: none of the following outcomes were reported: ROC, *c*-index, sensitivity, specificity, accuracy, recovery rate, accuracy rate, confusion matrix, *F*_1_-score, and calibration curve.

### Data Sources and Search Strategy

PubMed, Cochrane, Embase, and Web of Science were thoroughly retrieved up to September 3, 2023, using the form of MeSH (Medical Subjects Headings) + free term, without any restrictions on region or publication period. The specific search strategy is depicted in Multimedia Appendix 2.

### Study Selection and Data Extraction

Duplicates were excluded from the retrieved literature, and titles and abstracts were reviewed to delete obviously irrelevant studies. The full texts of the remaining studies were then downloaded and thoroughly read to determine the final included studies in the systematic review. A standard electronic data extraction spreadsheet was prepared prior to extracting data. The extracted data encompassed the title, first author, type of study, year of publication, author’s country, patient source, target event, number of cases of the target event, the total number of cases, number of training set cases, the total number of training set cases, method of validation set generation, number of events in the validation set, total number of cases in the validation set, type of models, and modeling variables.

Two researchers (HZ and LF) independently screened the literature and extracted data. Upon completion, their findings were cross-checked. A third reviewer (JH) was consulted for resolution in case of any dissents.

### Risk of Bias in Studies

The risk of bias in the eligible studies was appraised by two independent reviewers (HZ and LF) using the prediction model risk of bias assessment tool [16]. This tool is comprised of a large number of questions in four domains (participants, predictors, outcomes, and analysis), which reflect overall bias risk and applicability. The 4 domains involve 2, 3, 6, and 9 specific questions, respectively, and each question may be answered by yes or probably yes, no or probably not, or no information. Following the quality evaluation, a cross-check was carried out. In the event of any disputes, a third researcher (JH) was consulted for resolution.

### Synthesis Methods

In some of the original studies included in our research, there was not only 1 validation set. Therefore, the number of models included in the meta-analysis does not equal the number of studies. The meta-analysis of sensitivity and specificity was executed using a bivariate mixed-effects model [17]. Sensitivity and specificity were meta-analyzed as per the diagnostic 2×2 table. However, most included studies did not provide the diagnostic 2×2 table. In such cases, the following two approaches were used to calculate the diagnostic 2×2 table: (1) it was computed based on sensitivity, specificity, and precision, combined with the number of cases; and (2) sensitivity and specificity were extracted based on the optimal Youden index, and then combined with the number of cases for calculation. The meta-analysis was implemented using R (version 4.2.0; R Foundation for Statistical Computing).

## Results

### Study Selection

**Figure 1:**
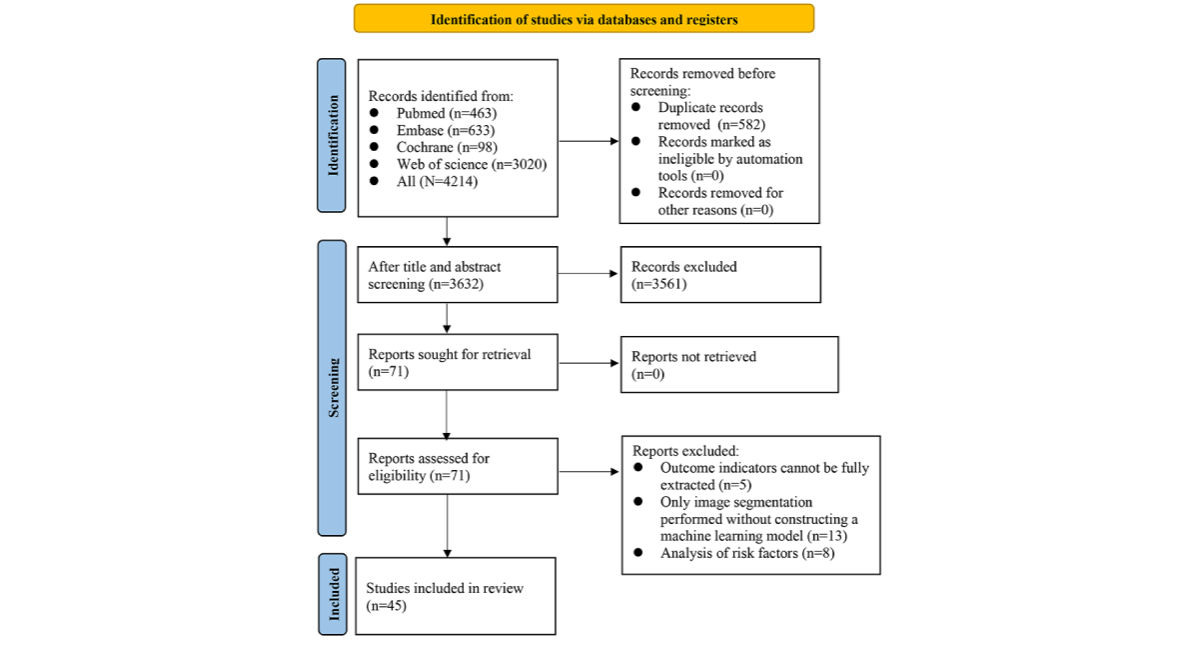
Flowchart of literature screening.

A total of 4214 records were retrieved from the databases, of which 582 were duplicates. After reading the titles and abstracts, 3561 studies unrelated to ML in high myopia were excluded, leaving 71 studies. Of these, 13 only conducted image segmentation without constructing ML models, 5 did not provide full extractable outcome indicators, and 8 analyzed risk factors. Ultimately, 45 studies were incorporated into this review. The literature screening process is depicted in Figure 1.

### Study Characteristics

The included studies were published from 2010 to 2023. Four of the studies [18-21] were about the prediction of high myopia, and the predicted variables were mainly derived from life characteristics, environmental and genetic factors, and routinely interpretable ocular clinical characteristics. Five of the studies [22-26] were about the diagnosis of high myopia, of which 1 study [22] also involved the diagnosis of pathological lesions of high myopia. Six studies focused on the diagnosis of high myopia-associated glaucoma [27-32]. Out of the included studies, 31 studies focused on the diagnosis of pathological myopia, primarily using optical coherence tomography and fundus imaging to construct artificial intelligence models. Of these, 26 studies [4,15,22,33-55] were based on DL (deep learning), while 5 studies [56-60] required manually coded ML for construction. Additionally, it was noted that in the 45 original studies, all 45 studies included binary classification tasks, with 9 studies [4,33,34,38,39,49,50,52,61] additionally incorporating multiclassification tasks. Regarding validation methods, 31 studies provided an external validation set, and 23 used a combination of internal and external validation sets. In terms of the generation method of validation set, 6 studies [23,24,34,40,47,59] used *k*-fold cross-validation, 29 [15,19-22,25-29,35-38,41,42,45,48-58,61] used random sampling, and 6 [4,18,32,33,44,60] applied a combination of *k*-fold cross-validation and random sampling. The detailed characteristics of the eligible studies are shown in Tables 1 and 2.

**Table 1:** Fundamental features of included studies.

First author	Year of publication	Country of authors	Study type	Patient source	Target events	Total number of cases
Tang et al [33]	2022	China, United States	Retrospective study	Multicenter	Diagnosis of pathologic myopia	1395 fundus photographs, 895 patients
Li et al [56]	2023	China	Nested case-control study	Single center	Diagnosis and prediction of pathological myopia	20,870 patients
Du et al [57]	2021	China	Retrospective study	Single center	Diagnosis of pathologic myopia	313 patients with high myopia and 457 eyes
Foo et al [18]	2023	Singapore	Prospective study	Multicenter	Prediction of high myopia	965 children with 1878 eyes and 7456 fundus photographs
Kim et al [58]	2021	Korea	Retrospective study	Multicenter	Diagnosis of pathologic myopia	860 eyes
Zhang et al [59]	2013	Singapore	Retrospective study	Registry database	Diagnosis of pathologic myopia	2258 patients
Zhu et al [34]	2023	China	Retrospective study	Single center	Diagnosis of pathologic myopia	6078 photographs
Wu et al [35]	2022	China	Retrospective study	Single center	Diagnosis of pathologic myopia	1853 photographs
Ye et al [36]	2021	China	Retrospective study	Single center	Diagnosis of pathologic myopia	1041 patients with pathologic myopia and with 2342 eligible OCT^a^ macular images
Wang et al [37]	2023	China	Retrospective study	Single center	Diagnosis of pathologic myopia	7606 patients with 10,347 fundus photographs
Wang et al [19]	2022	China	Prospective, longitudinal, observational study	Wenzhou large-scale survey	Prediction of myopia and high myopia	15,765 patients
Wan et al [4]	2021	China	Retrospective study	Single center	Diagnosis of pathologic myopia	858 photographs
Wan et al [38]	2023	China	Retrospective study	Single center	Diagnosis of pathologic myopia	1750 photographs
Tan et al [22]	2021	Singapore	Retrospective multicohort study	Multicenter + registry database	Diagnosis of high myopia + pathological myopia	125,421 patients with 251,349 photographs
Sun et al [39]	2023	China	Retrospective multicohort study	Multicenter + registry database	Diagnosis of pathologic myopia	1514 fundus photographs
Sogawa et al [40]	2020	Japan	Retrospective study	Single center	Diagnosis of pathologic myopia	910 patients with 910 images
Du et al [41]	2022	Japan	Retrospective study	Single center	Diagnosis of pathologic myopia	1327 patients with 2400 high myopia eyes and 9176 OCT images
Hou et al [60]	2023	China	Prospective cohort study	Single center	Diagnosis of pathologic myopia	576 patients
Li et al [52]	2022	China	Retrospective cohort study	Multicenter	Pathologic myopia	29,230 patients with 57,148 fundus photographs
Li et al [27]	2021	China	Case-control study	Multicenter	Diagnosis of glaucoma in high myopia	2731 participants with 2731 eyes
Chen et al [20]	2019	China	Prospective study	Single center	Prediction of high myopia	1063 patients
Choi et al [23]	2021	Korea	Retrospective study	Single center	Prediction of high myopia	492 patients with 690 eyes
Cui et al [42]	2021	China, Taiwan	Retrospective study	Registry database	Diagnosis of pathologic myopia	800 images
Guan et al [24]	2023	China	Retrospective study	Multicenter	Prediction of high myopia	1,285,609 participants
He et al [61]	2022	China	Retrospective study	Multicenter	Diagnosis of pathologic myopia	2866 patients with 3945 OCT images
Hemelings et al [15]	2021	Belgium	Retrospective study	Registry database	Diagnosis of pathologic myopia	1200 photographs
Rauf et al [44]	2021	Pakistan	Retrospective study	Registry database	Diagnosis of pathologic myopia	840 photographs
Park et al [45]	2022	Korea	Retrospective study	Single center	Diagnosis of pathologic myopia	367 eyes
Lu et al [46]	2021	China	Retrospective study	Single center	Diagnosis of pathologic myopia and diagnosis of pathologic myopia	17,330 photographs 17,330 photographs
Lu et al [47]	2021	China	Retrospective study	Multicenter	Diagnosis of pathologic myopia	32,419 patients with 37,659 images
Liu et al [54]	2010	Singapore	Retrospective study	Single center	Pathologic myopia	80 photographs
Li et al [48]	2022	China, United States	Retrospective study	Single center	Diagnosis of pathologic myopia	1139 patients with 5917 images
Lee et al [28]	2023	Korea	Retrospective study	Single center	Diagnosis of glaucoma in high myopia	260 eyes and 260 images
Kim et al [29]	2023	Korea	Retrospective study	Single center	Diagnosis of glaucoma in high myopia	2607 eyes
Jeong et al [30]	2023	Korea	Retrospective cross-sectional study	Single center	Diagnosis of glaucoma in high myopia	274 patients
Huang et al [21]	2022	China	Case-control study	Single center	Prediction of high myopia	1298 patients
Huang et al [49]	2023	China, United Kingdom	Retrospective study	Single center	Diagnosis of pathologic myopia	1131 patients with 3441 images
Du et al [50]	2021	Japan	Retrospective study	Single center	diagnosis of pathologic myopia	4432 eyes and 7020 images
Crincoli et al [51]	2023	Italy	Case-control study	Multicenter	diagnosis of pathologic myopia	84 patients with 84 eyes and 252 photographs
Asaoka et al [31]	2014	Japan	Case-control study	Multicenter	Diagnosis of glaucoma in high myopia	242 patients and 242 eyes
Bowd et al [32]	2023	United States, Germany	Retrospective study	Single center	Diagnosis of glaucoma in high myopia	593 eyes
Zhao et al [25]	2022	China	Retrospective study	Single center	Prediction of high myopia	546 patients
Liu et al [53]	2010	Singapore	Retrospective study	Single center	Diagnosis of pathologic myopia	80 photographs
Dai et al [26]	2020	China	Retrospective study	Single center	Prediction of high myopia	319 patients with 932 images
Baid et al [55]	2019	India	Retrospective study	Registry database	Diagnosis of pathologic myopia	481 photographs

^a^OCT: optical coherence tomography.

**Table 2:** Fundamental features of included studies.

Total number of cases in training set	Generation of validation set	Total number of cases in validation set	Total number of cases in test set	Model type	Modeling variables
727 fundus photographs	5-fold cross-validation + random sampling	238 fundus photographs	238 fundus photographs	DL^a^	Fundus photographs
2069 patients	Random sampling	1382 patients	Unclear	ACP^b^, ML^c^	Clinical features
319 eyes	Random sampling	138 eyes	Unclear	ML-based radiomics analysis method	Fundus photographs
769 children with 1502 eyes and 5945 photographs	Internal validation (5-fold cross-validation + random sampling) + multicenter external validation	196 children with 376 eyes and 1511 fundus photographs	99 children with 189 eyes and 821 photographs	DL	Fundus photographs + clinical features
602 eyes	Random sampling	258 eyes	unclear	SVM^d^, ML	Fundus photographs
2258 patients	Stratified 20-fold cross-validation	unclear	unclear	SVM, ML	SNP^e^ + clinical features + fundus photographs
4252 photographs	Stratified 20-fold cross-validation	unclear	1826 photographs	DL	Fundus photographs
1483 photographs	Random sampling	unclear	370 photographs	DL	Fundus photographs
1874 photographs	Internal validation (random sampling) + external validation（multicenter）	468 photographs	450 photographs	DL	Fundus photographs
5003 patients with 7389 photographs	Random sampling	775 patients with 821 photographs	1828 patients with 2137 photographs	DL	Fundus photographs
11,350 patients	Internal validation (random sampling) + external validation(prospective)	4415 patients	6168 patients (prognostic cohort)	LR^f^, GBDT^g^, NN^h^	Clinical features
758 photographs	5-fold cross-validation + random sampling	100 photographs	Unclear	DL	Fundus photographs
1402 photographs	Random sampling	174 photographs	174 photographs	DL	Fundus photographs
226,686 photographs	Internal validation (random sampling) + external validation（multicenter）	11,303 photographs	213,475 photographs	DL	Fundus photographs
400 fundus photographs	Multicenter	400 fundus photographs	714 fundus photographs	DL	Fundus photographs
Unclear	5-fold cross-validation	Unclear	Unclear	DL	Fundus photographs
7865 photographs	random sampling	1311 photographs	Unclear	DL	Fundus photographs
516 patients	10-fold cross-validation + random sampling	60 patients	Unclear	XGBoost^i^, SVM, LR	Clinical features + metabolic characteristics
29,213 photographs	Internal validation (random sampling) + external validation（multicenter）	7302 photographs	16,554 photographs	DCNN^j^, DL	Fundus photographs
2223 participants with 2223 eyes	Random sampling	508 participants with 508 eyes	Unclear	FCN^k^	OCT^l^ images + clinical features
638 patients	Random sampling	425 patients	Unclear	LR	Genetic factors + clinical features
434 patients with 600 eyes and 1200 images	5-fold cross-validation	Unclear	58 patients with 90 eyes and 180 images	CNN^m^, DL	OCT images
400 images	Random sampling	200 images	200 images	DL	Fundus photographs
1600 participants	Internal validation (5-fold cross-validation)	Unclear	400 patients	RF^n^, LR, SVM	Clinical features
2380 images	Random sampling	680 photographs	340 photographs	DL	OCT images
400 photographs	Random sampling	400 photographs	400 photographs	DL	Fundus photographs
400 photographs	10-fold cross-validation + random sampling	40 photographs	400 photographs	DL	Fundus photographs
293 eyes	random sampling	37 eyes	37 eyes	DL	3D OCT images
11,502 photographs	Unclear	3284 photographs	1642 photographs	DL	Fundus photographs
2457 photographs	Unclear	707 photographs	372 photographs	DL	Fundus photographs
32,010 images	Internal validation (5-fold cross-validation) + external validation (multicenter)	Unclear	732 patients with 1000 images	DL	Fundus photographs
40 photographs	Random sampling	Unclear	40 photographs	SVM, DL	Fundus photographs
838 patients with 4338 images	Internal validation (random sampling) + external validation (prospective)	210 patients with 1167 photographs	91 patients with 174 eyes and 412 photographs	DL	OCT macular images
165 images	Random sampling	46 photographs	49 photographs	DL	OCTA^o^ and OCT images
1416 eyes	Internal validation (random sampling) + external validation	471 eyes	720 eyes	DL	OCT images
Unclear	Unclear	Unclear	Unclear	Decision tree	OCT images
325 patients	Random sampling	973 patients	Unclear	DL	Genetic + clinical features
2264 images	Internal validation (random sampling) + external validation (prospective)	501 photographs	604 photographs	DL	OCT images
4140 photographs	Random sampling	1036 photographs	1844 photographs	DL	Fundus photographs
176 photographs	Random sampling	25 photographs	51 photographs	DL	OCT images
Unclear	Unclear	Unclear	Unclear	RF	HRT parameters
347 eyes	5-fold cross-validation + random sampling	87 eyes	159 eyes	CNN	OCT images
928 fundus photographs	Random sampling	232 photographs	Unclear	DL	Fundus photographs
40 photographs	Random sampling	Unclear	40 photographs	SVM or DL	Fundus photographs
792 photographs	Random sampling	Unclear	140 photographs	DL	Fundus photographs
374 photographs	Random sampling	80 photographs	27 photographs	CNN	Fundus photographs

^a^DL: deep learning.

^b^ACP: algorithm of conditional probability.

^c^ML: machine learning.

^d^SVM: support vector machine.

^e^SNP: single nucleic polymorphism.

^f^LR: logistic regression.

^g^GBDT: gradient boosted decision tree.

^h^NN: neural network.

^i^XGBoost: extreme gradient boosting

^j^DCNN: deep convolutional neural networks

^k^FCN: fully connected network

^l^OCT: optical coherence tomography.

^m^CNN: convolutional neural networks

^n^RF: random forest

^o^OCTA: optical coherence tomography angiography.

### Risk of Bias in Studies

This review incorporated 67 models. There were 36 retrospective studies [4,15,22-26,28-30,32-42,44-50,52-55,57-59,61] that constructed 39 models, indicating a high bias in the choosing of study participants. Five case-control studies [21,27,31,51,56] constructed 13 models, also showing high bias in the selection of study participants. Since the predictors were evaluated in the context of a known outcome in the case-control studies, there was a high bias in the assessment of predictive factors. Twelve studies [19,20,23,24,27,30,31,56-60] constructed 22 models based on manually coded ML, with a high bias in predictive factors. In terms of statistical analysis, 2 studies [21,45] with 5 models did not meet the requirement of having an event per variable>20%, indicating a high risk of bias. In the statistical analysis, 32 models in 34 studies [4,15,18,21-23,25,26,28,29,32-42,44-55,61] could not estimate event per variable due to the use of the DL method. Additionally, 10 studies [19,20,24,27,30,31,56,58-60] with 29 models in ML did not report on the complexity of the data, rendering it difficult to determine their bias risk. Five studies [20,27,30,31,60] with 11 models were identified as having a high risk of bias in statistical analysis because they did not perform cross-validation to adjust the stability of models with different parameters. In summary, in terms of research participants, 14 models had a low risk of bias; 52 models had a high risk of bias, and 1 model had an unclear risk of bias. In terms of predictors, 37 models had a low risk of bias and 30 models had a high risk of bias. In terms of outcomes, all 67 models had a low risk of bias. In terms of statistical analysis, 3 models had a low risk of bias, 16 models had a high risk of bias, and 48 models had an unclear risk of bias.

### Meta-Analysis of ML for Binary Classification Tasks

#### Pathological Myopia

**Figure 2:**
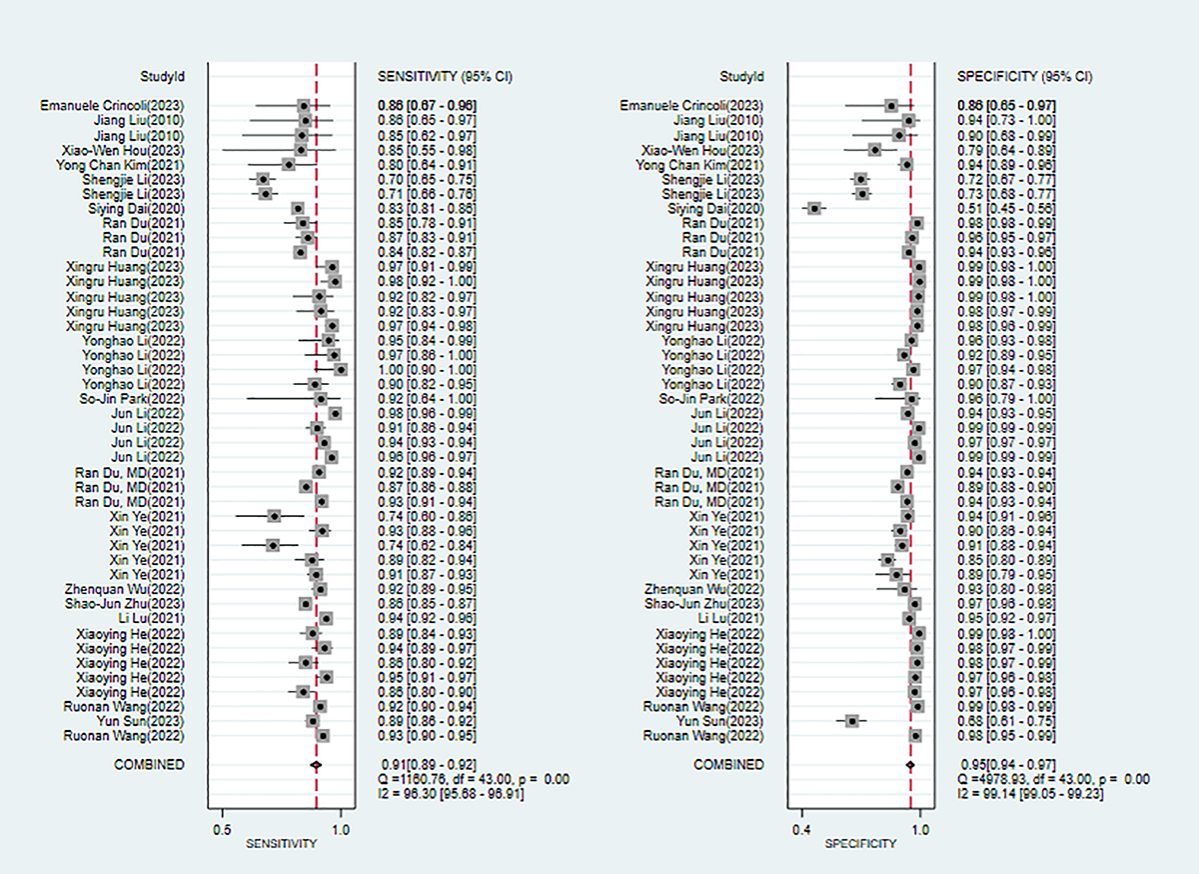
Forest plot for the meta-analysis of sensitivity and specificity of machine learning in detecting pathological myopia [26,34-37,39,41,45,47-54,56,58,60,61]. Note: the pooled sensitivity and specificity of 44 models from 20 machine learning studies on the diagnosis of pathological myopia were 0.91 (95% CI 0.89-0.92) and 0.95 (95% CI 0.94-0.97), respectively.

Twenty studies [26,34-37,39,41,45,47-54,56,58,60,61] reported ML for diagnosing pathological myopia. Modeling algorithms included algorithms of conditional probability, support vector machines (SVMs), logistic regression (LR), extreme gradient boosting, convolutional neural networks (CNNs), and deep convolutional neural networks (DCNNs). The overall sensitivity, specificity, positive likelihood ratio (PLR), negative likelihood ratio (NLR), diagnostic odds ratio (DOR), and summary receiver operating characteristic (SROC) were 0.91 (95% CI 0.89-0.92), 0.95 (95% CI 0.94-0.97), 19.7 (95% CI 13.8-28.2), 0.10 (95% CI 0.08-0.12), 201 (95% CI 122-331), and 0.97 (95% CI 0.95-0.98), respectively. The Deek funnel plot indicated no substantial evidence of publication bias in the included ML models. Assuming that the prior probability of pathological myopia was 20% if the result of ML was pathological myopia, then the probability of true pathological myopia would be 83%. If the result of ML was nonpathological myopia, then the probability of true pathological myopia would be 2% (ie, the probability of true nonpathological myopia was 98%; Figure 2 and Figures S1-S3 in Multimedia Appendix 3).

**Figure 3:**
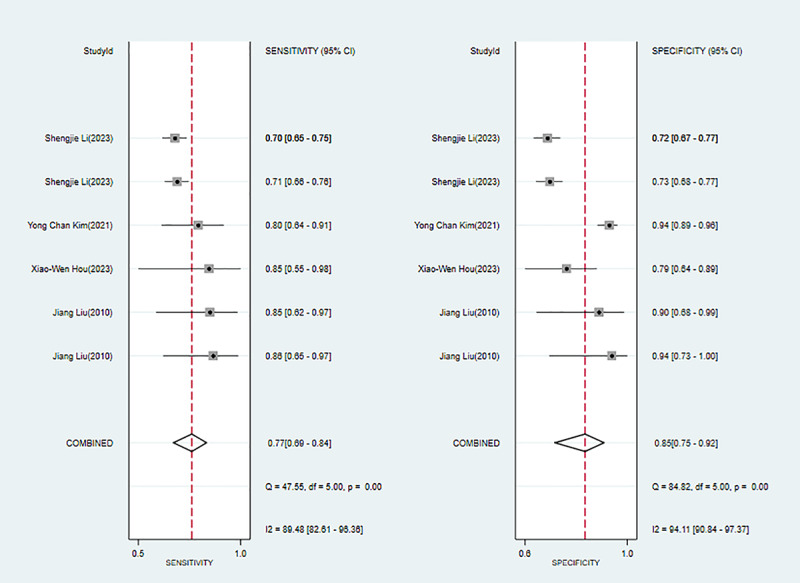
Forest plot for the meta-analysis of sensitivity and specificity of conventional machine learning (non-deep learning) in detecting pathological myopia [53,54,56,58,60]. Note: the pooled sensitivity and specificity of 6 models from 5 conventional machine learning (non-deep learning) studies on the diagnosis of pathological myopia were 0.77 (95% CI 0.69-0.84) and 0.85 (95% CI 0.75-0.92), respectively.

Five studies [53,54,56,58,60] reported conventional ML (non-DL) for diagnosing pathological myopia. Modeling algorithms included algorithms of conditional probability, SVM, extreme gradient boosting, and LR. The overall sensitivity, specificity, PLR, NLR, DOR, and SROC curve were 0.77 (95% CI 0.69-0.84), 0.85 (95% CI 0.75-0.92), 5.2 (95% CI 2.8-9.8), 0.27 (95% CI 0.18-0.39), 20 (95% CI 7-51), and 0.86 (95% CI 0.75-0.92), respectively. The Deek funnel plot indicated the presence of publication bias in the conventional ML (non-DL) models. Assuming that the prior probability of pathological myopia for conventional ML (non-DL) was 20% if the result of conventional ML (non-DL) was pathological myopia, then the probability of true pathological myopia would be 57%. If the result of conventional ML (non-DL) was nonpathological myopia, then the probability of true pathological myopia would be 6% (ie, the probability of true nonpathological myopia was 94%; Figure 3 and Figures S4-S6 in Multimedia Appendix 3).

**Figure 4:**
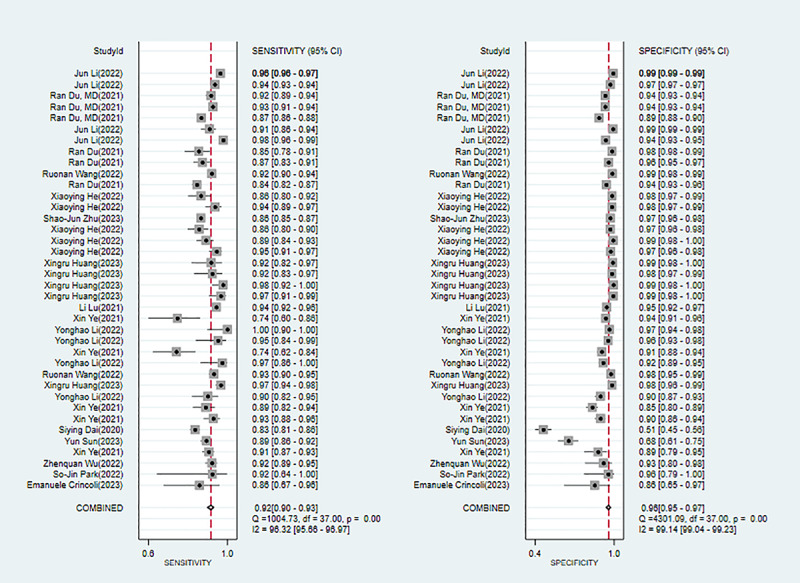
Forest plot for the meta-analysis of sensitivity and specificity of deep learning in detecting pathological myopia [26,34-37,39,41,45,47-52,61]. Note: the pooled sensitivity and specificity of 38 models from 15 deep learning studies on the diagnosis of pathological myopia were 0.92 (95% CI 0.90-0.93) and 0.96 (95% CI 0.95-0.97), respectively.

Fifteen studies [26,34-37,39,41,45,47-52,61] mentioned DL for diagnosing pathological myopia. Modeling algorithms included CNN and DCNN. The overall sensitivity, specificity, PLR, NLR, DOR, and SROC were 0.92 (95% CI 0.90-0.93), 0.96 (95% CI 0.95-0.97), 23.7 (95% CI 16.5-34.0), 0.09 (95% CI 0.07-0.11), 271 (95% CI 168-437), and 0.97 (95% CI 0.95-0.98), respectively. The Deek funnel plot revealed no remarkable publication bias in the DL models. Assuming that the prior probability of pathological myopia for DL was 20% if the result of DL was pathological myopia, then the probability of true pathological myopia would be 86%. If the result of DL was nonpathological myopia, then the probability of true pathological myopia would be 2% (ie, the probability of true nonpathological myopia was 98%; Figure 4 and Figures S7-S9 in Multimedia Appendix 3).

#### High Myopia

**Figure 5:**
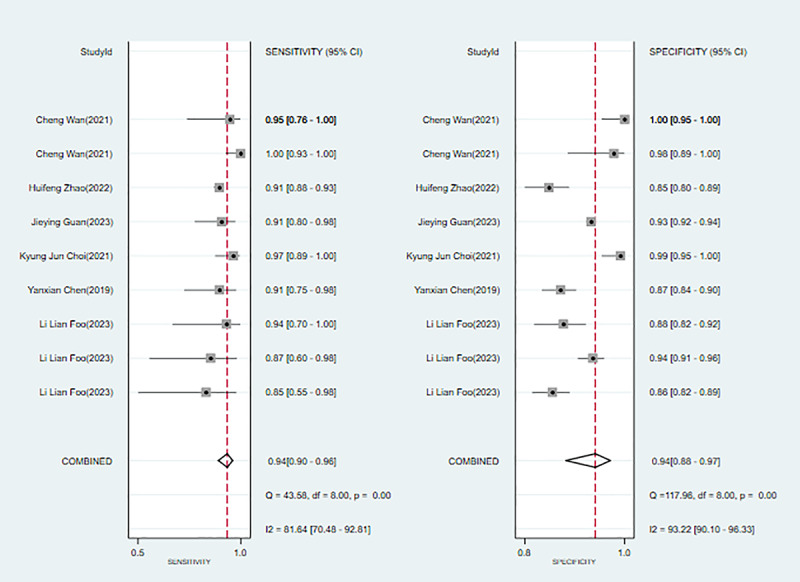
Forest plot for the meta-analysis of sensitivity and specificity of machine learning in detecting high myopia [4,18,20,23-25]. Note: the pooled sensitivity and specificity of 9 models from 6 machine learning studies on the diagnosis and prediction of high myopia were 0.94 (95% CI 0.90-0.96) and 0.94 (95% CI 0.88-0.97), respectively.

Six studies [4,18,20,23-25] discussed ML for diagnosing and forecasting high myopia. Modeling algorithms included DCNN, CNN, LR, SVM, random forest (RF), and linear mixed models. The sensitivity, specificity, PLR, NLR, DOR, and SROC were 0.94 (95% CI 0.90-0.96), 0.94 (95% CI 0.88-0.97), 16.2 (95% CI 7.7-33.8), 0.06 (95% CI 0.04-0.11), 255 (95% CI 79-822), and 0.98 (95% CI 0.96-0.99), respectively. The Deek funnel plot indicated no substantial evidence of publication bias in the included ML models. Assuming that the prior probability of high myopia for ML was 20% if the result of ML was high myopia, then the probability of true high myopia would be 80%. If the result of ML was non-high myopia, then the probability of true high myopia would be 2% (ie, the probability of true non-high myopia was 98%; Figure 5 and Figures S10-S12 in Multimedia Appendix 3).

Three studies [4,23,25] focused on diagnosing high myopia, while 3 studies [18,20,24] focused on predicting high myopia. Due to the limited number of studies included, we did not perform a meta-analysis for the diagnostic and prediction tasks. In the validation sets of the diagnostic tasks, sensitivity ranged from 0.91 to 1.00 and specificity ranged from 0.85 to 1.00, while in the validation sets of the prediction tasks, these values were 0.85-0.94 and 0.86-0.94, respectively. We found that both diagnostic and prediction tasks demonstrated highly favorable performance.

#### High Myopia–Associated Glaucoma

**Figure 6:**
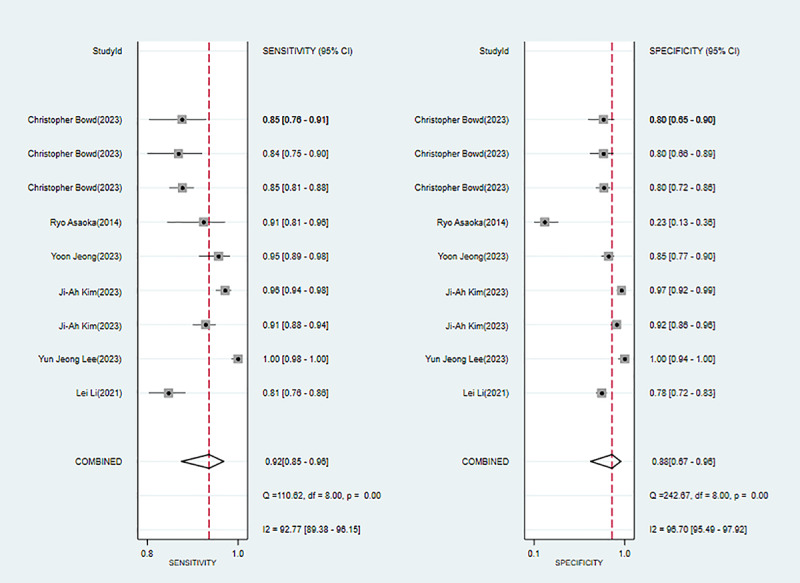
Forest plot for the meta-analysis of sensitivity and specificity of machine learning in detecting high myopia-associated glaucoma [27-32]. Note: the pooled sensitivity and specificity of 9 models from 6 machine learning studies on the diagnosis of high myopia-associated glaucoma were 0.92 (95% CI 0.85-0.96) and 0.88 (95% CI 0.67-0.96), respectively.

Six studies [27-32] mentioned ML for diagnosing high myopia-associated glaucoma. Modeling algorithms included Lagrange multiplier, fully connected network, radial basis function network, decision tree, RF, and CNN. The sensitivity, specificity, PLR, NLR, DOR, and SROC curve were 0.92 (95% CI 0.85-0.96), 0.88 (95% CI 0.67-0.96), 7.6 (95% CI 2.4-23.8), 0.09 (95% CI 0.04-0.20), 84 (95% CI 13-555), and 0.96 (95% CI 0.94-0.97), respectively. The Deek funnel plot indicated no substantial evidence of publication bias in the included ML models. Assuming that the prior probability of high myopia–associated glaucoma was 20% if the result of ML was high myopia-associated glaucoma, then the probability of true high myopia–associated glaucoma would be 65%. If the result of ML was non-high myopia–associated glaucoma, then the probability of true high myopia–associated glaucoma would be 2% (ie, the probability of true non-high myopia–associated glaucoma was 98%; Figure 6 and Figures S13-S15 in Multimedia Appendix 3).

### Review of ML for Multiclassification Tasks

Out of the included studies, 9 [4,33,34,38,39,49,50,52,61] used ML for multiclassification tasks. Due to significant variations in the diagnostic differences across these multiclassification tasks, a quantitative analysis was not feasible. Five studies [33,34,38,39,50] focused on fundus images–based DL to detect different types of myopic atrophy maculopathy in high myopia, with an accuracy ranging from 88% to 97%. Two studies [49,61] used optical coherence tomography (OCT) image–based DL to detect different types of myopic traction maculopathy in high myopia, with an accuracy ranging from 91% to 96%. One study [4] used fundus image–based DL to differentiate between normal, low-risk high myopia, and high-risk high myopia, with an accuracy of 99%. One study [52] applied fundus image–based DL to distinguish between normal, fundus tessellation, and pathologic myopia, with an accuracy of 94%, as illustrated in Table 3.

**Table 3:** Results of machine learning for multiclassification tasks.

First author	Year	Diagnostic purpose	Types of artificial intelligence	Modeling variables	Generation of validation set	Accuracy rate, %
Tang et al [33]	2022	Classification of atrophic macular lesions in myopic	CNNs^a^; DL^b^	Fundus photographs	5-fold cross-validation + random sampling	94
Zhu et al [34]	2023	Classification of atrophic macular lesions in myopic	Neural network; DL	Fundus photographs	Stratified 20-fold cross-validation	90
Wan et al [4]	2021	Normal, low, and high risk of high myopia	DCNNs^c^; DL	Fundus photographs	5-fold cross-validation + random sampling	99
Wan et al [38]	2023	Classification of atrophic macular lesions in myopic	DL	Fundus photographs	Random sampling	95-97
Sun et al [39]	2023	Classification of atrophic macular lesions in myopic	DL	Fundus photographs	External validation (multicenter)	89.2
Li et al [52]	2022	Differential diagnosis of normal, leopard print fundus, and pathological myopia	DCNN; DL	Fundus photographs	Internal validation (random sampling) + external validation (multicenter)	94
He et al [61]	2022	Differential diagnosis of tractive macular degeneration and neovascular macular degeneration in high myopia, and others	DL	OCT^d^ images	Random sampling	91-96
Huang et al [49]	2023	Classification of tractive macular degeneration in high myopia	DL	OCT images	Internal validation (random sampling) + external validation (prospective)	96
Du et al [50]	2021	Classification of atrophic macular lesions in myopic	DL	Fundus photographs	Random sampling	88

^a^CNN: convolutional neural network.

^b^DL: deep learning.

^c^DCNN: deep convolutional neural network.

^d^OCT: Optical Coherence Tomography.

## Discussion

### Summary of the Main Findings

This study comprehensively described the accuracy of ML in detecting high myopia, high myopia-associated glaucoma, and pathologic myopia. ML demonstrated exceptionally favorable performance in detecting high myopia, while DL was highly accurate in diagnosing pathologic myopia.

### Comparison With Previous Reviews

Previous studies have also explored the detection accuracy of ML in this field. A systematic review has reported that fundus image– or OCT image–based DL can effectively diagnose and classify myopic maculopathy. Additionally, ML examination of the optic disc area can detect myopic maculopathy that may not be easily identified during clinical examination [14]. A recent meta-analysis based on only 11 studies evaluated the performance of DL in identifying pathological myopia based on fundus images. The SROC, specificity, and sensitivity were found to be 0.9905, 0.959 (95% CI 0.955-0.962), and 0.965 (95% CI 0.963-0.966), respectively [62]. In the previous meta-analysis, the 11 original studies all constructed fundus images-based DL models, and studies on conventional ML (non-DL) were not incorporated. The number of studies included in our review was further expanded, with a total of 20 studies on the performance of ML in diagnosing pathological myopia. Moreover, subgroup analysis was executed between conventional ML (non-DL) and DL. Our finding also indicated that DL demonstrated exceptionally favorable efficiency in detecting pathological myopia.

As the understanding of the etiology of myopia deepens, growing evidence reveals risk factors for the onset or progression of myopia, including age, sex, parental myopia, susceptibility genes, and outdoor activities. For high myopia, early prediction appears to be more beneficial. Among the included studies, one incorporated 135 myopia-related single nucleotide polymorphisms to forecast the progression and onset of high myopia. ML for the prediction of high myopia was mainly based on genetic factors, environmental factors, and ocular clinical characteristics. ML showed an SROC of 0.96, sensitivity of 0.91, and specificity of 0.87, respectively [20], suggesting that ML methods can effectively identify high-risk individuals with high myopia, thus effectively preventing this condition, especially in minors.

Glaucoma is a significant contributor to irreversible vision impairment and blindness all over the world. A 10-year study in Chinese individuals over the age of 40 years found that every 1 mm increase in axial length increased the risk of open-angle glaucoma by 1.72 times. In comparison to emmetropic and hyperopic eyes, highly myopic eyes had a 7.3 times higher risk of developing open-angle glaucoma [63]. Due to the changes in retinal structure caused by myopia, diagnosing glaucoma in myopic patients, especially those with high myopia, is challenging. Six studies were included to evaluate the diagnosis of high myopia glaucoma. Of them, 3 studies [28,29,32] used fundus OCT image-based DL techniques, while the remaining 3 [27,30,31] used non-DL ML (Lagrange multiplier, fully connected network, radial basis function network, decision tree, RF) approaches using OCT parameters, Heidelberg Retina Tomograph parameters, and ocular biometric parameters of patients. The findings indicated that ML yielded highly promising results in the detection of high myopia glaucoma.

It was also noted that different ML methods, conventional ML and DL, showed significant differences in their ability to identify positive or outcome events. Conventional ML is often used to construct models with interpretable clinical features. Lately, various image-based ML methods have emerged. However, a significant challenge in this context is the requirement for manual annotation to facilitate ML. From this standpoint, manual annotation poses a formidable barrier to effectively mitigating the risk of bias. DL, on the other hand, enables intelligent processing of medical images and has been widely applied in various fields, including detecting diabetic retinopathy [8-10], retinopathy of prematurity [64,65], age-related macular degeneration [10], and glaucoma [11-13]. With the rapid development of ML, imaging data are increasingly becoming a valuable source for medical analysis. Multiple studies have demonstrated that images from various sources, including fundus images [66], external eye appearance [67], and refractive images [68], can effectively estimate a patient’s spherical refractive error, indicating the potential of imaging data in predicting the risk of myopia. This study also finds that image-based DL is more accurate than conventional ML, providing a theoretical basis for the creation of future intelligent tools.

Additionally, the dataset used in ML demands considerable attention. Many studies are hampered by a limited number of cases, raising concerns about the robustness of the findings. Additionally, validation methods often depend heavily on internal validation, which may not fully capture the model’s generalizability. Hence, incorporating comprehensive patient data is essential for building a robust large-scale database, which will enable the development of ML models that are applicable to a broader population. Among the studies included, 7 [15,22,39,42,44,55,59] established ML models based on publicly available large databases.

### Limitations

Although our review includes a larger number of studies than previous meta-analyses and provides an evidence-based basis for subsequent studies, this study has limitations. First, there were few studies on the prediction of high myopia, which limits the interpretation of our results, and clinically interpretable variables for predicting high myopia were not explained. Second, we did not conduct a subgroup analysis on the type of ML (conventional ML vs DL) owing to the insufficient number of included studies based on high myopia glaucoma and high myopia. Third, the majority of the models included in this study were assessed as having a high risk of bias, which may impact the interpretation of our results. Most included studies adopted a retrospective design, which might lead to selection bias.

### Conclusions

In conclusion, this study comprehensively reviews and meta-analyzes the performance of ML in the diagnosis and prediction of high myopia, high myopia-associated glaucoma, and pathological myopia, providing valuable guidance and references for future research. Challenges exist within the emerging field of myopia prediction. With the development of new analytical methods and the accumulation of real medical datasets, future research holds the promise of improving the prediction of myopia onset and progression. This advancement brings us closer to the ultimate goal of identifying high-risk individuals promptly and implementing targeted interventions in clinical practice.

## Appendix

Multimedia Appendix 1 PRISMA (Preferred Reporting Items for Systematic Reviews and Meta-Analyses) checklist.

Multimedia Appendix 2 Literature search strategy.

Multimedia Appendix 3 Additional figures.

## Abbreviations

CNN: convolutional neural network
DCNN: deep convolutional neural network
DL: deep learning
DOR: diagnostic odds ratio
LR: logistic regression
MeSH: Medical Subjects Headings
ML: machine learning
NLR: negative likelihood ratio
OCT: optical coherence tomography
PLR: positive likelihood ratio
PRISMA: Preferred Reporting Items for Systematic Reviews and Meta-Analyses
RF: random forest
SROC: summary receiver operating characteristic
SVM: support vector machine

## Data Availability

All data generated or analyzed during this study are included in this published article. Further inquiries can be directed to the corresponding author.
