# Supplementary material for: Machine Learning Approaches in High Myopia: Systematic Review and Meta-Analysis
Source: J Med Internet Res. 2025 Jan 3;27:e57644. doi: 10.2196/57644 (PMC11748443; doi:10.2196/57644)
Supplement: Multimedia Appendix 2 [file jmir_v27i1e57644_app2.docx]

**Table S2**: Literature search strategy.

**1.Pubmed**

| Search number | Query | Results |
| --- | --- | --- |
| #1 | "Myopia"[Mesh] | 21,271 |
| #2 | ((((((((((((myopia[Title/Abstract]) OR (Myopias[Title/Abstract])) OR (Nearsightedness[Title/Abstract])) OR (Nearsightednesses[Title/Abstract])) OR (myopy[Title/Abstract])) OR (near-sightedness[Title/Abstract])) OR (short-sightedness[Title/Abstract])) OR (Shortsightedness[Title/Abstract])) OR (Refractive errors[Title/Abstract])) OR (Refractive error[Title/Abstract])) OR (Short sight[Title/Abstract])) OR (Near sight[Title/Abstract])) OR (Myopic[Title/Abstract]) | 33,301 |
| #3 | ("Myopia"[Mesh]) OR (((((((((((((myopia[Title/Abstract]) OR (Myopias[Title/Abstract])) OR (Nearsightedness[Title/Abstract])) OR (Nearsightednesses[Title/Abstract])) OR (myopy[Title/Abstract])) OR (near-sightedness[Title/Abstract])) OR (short-sightedness[Title/Abstract])) OR (Shortsightedness[Title/Abstract])) OR (Refractive errors[Title/Abstract])) OR (Refractive error[Title/Abstract])) OR (Short sight[Title/Abstract])) OR (Near sight[Title/Abstract])) OR (Myopic[Title/Abstract])) | 37,854 |
| #4 | "Machine Learning"[Mesh] | 59,499 |
| #5 | (((((((((((((((((((((((((machine learning[Title/Abstract]) OR (Transfer Learning[Title/Abstract])) OR (Deep learning[Title/Abstract])) OR (Ensemble Learning[Title/Abstract])) OR (artificial intelligence[Title/Abstract])) OR (random forest[Title/Abstract])) OR (neural network[Title/Abstract])) OR (neural networks[Title/Abstract])) OR (K-Nearest Neighbor[Title/Abstract])) OR (CNN[Title/Abstract])) OR (AlexNet[Title/Abstract])) OR (VGGNet[Title/Abstract])) OR (ResNet[Title/Abstract])) OR (GoogLeNet[Title/Abstract])) OR (Support vector machine[Title/Abstract])) OR (SVM[Title/Abstract])) OR (Gradient Boosting Machine[Title/Abstract])) OR (Nomogram[Title/Abstract])) OR (XGBoost[Title/Abstract])) OR (Adaboost[Title/Abstract])) OR (Decision tree[Title/Abstract])) OR (Naive Bayesian[Title/Abstract])) OR (Multilayer perceptron[Title/Abstract])) OR (Bayesian network[Title/Abstract])) OR (Prediction model[Title/Abstract])) OR (Risk model[Title/Abstract]) | 296,539 |
| #6 | ("Machine Learning"[Mesh]) OR ((((((((((((((((((((((((((machine learning[Title/Abstract]) OR (Transfer Learning[Title/Abstract])) OR (Deep learning[Title/Abstract])) OR (Ensemble Learning[Title/Abstract])) OR (artificial intelligence[Title/Abstract])) OR (random forest[Title/Abstract])) OR (neural network[Title/Abstract])) OR (neural networks[Title/Abstract])) OR (K-Nearest Neighbor[Title/Abstract])) OR (CNN[Title/Abstract])) OR (AlexNet[Title/Abstract])) OR (VGGNet[Title/Abstract])) OR (ResNet[Title/Abstract])) OR (GoogLeNet[Title/Abstract])) OR (Support vector machine[Title/Abstract])) OR (SVM[Title/Abstract])) OR (Gradient Boosting Machine[Title/Abstract])) OR (Nomogram[Title/Abstract])) OR (XGBoost[Title/Abstract])) OR (Adaboost[Title/Abstract])) OR (Decision tree[Title/Abstract])) OR (Naive Bayesian[Title/Abstract])) OR (Multilayer perceptron[Title/Abstract])) OR (Bayesian network[Title/Abstract])) OR (Prediction model[Title/Abstract])) OR (Risk model[Title/Abstract])) | 301,665 |
| #7 | (("Myopia"[Mesh]) OR (((((((((((((myopia[Title/Abstract]) OR (Myopias[Title/Abstract])) OR (Nearsightedness[Title/Abstract])) OR (Nearsightednesses[Title/Abstract])) OR (myopy[Title/Abstract])) OR (near-sightedness[Title/Abstract])) OR (short-sightedness[Title/Abstract])) OR (Shortsightedness[Title/Abstract])) OR (Refractive errors[Title/Abstract])) OR (Refractive error[Title/Abstract])) OR (Short sight[Title/Abstract])) OR (Near sight[Title/Abstract])) OR (Myopic[Title/Abstract]))) AND (("Machine Learning"[Mesh]) OR ((((((((((((((((((((((((((machine learning[Title/Abstract]) OR (Transfer Learning[Title/Abstract])) OR (Deep learning[Title/Abstract])) OR (Ensemble Learning[Title/Abstract])) OR (artificial intelligence[Title/Abstract])) OR (random forest[Title/Abstract])) OR (neural network[Title/Abstract])) OR (neural networks[Title/Abstract])) OR (K-Nearest Neighbor[Title/Abstract])) OR (CNN[Title/Abstract])) OR (AlexNet[Title/Abstract])) OR (VGGNet[Title/Abstract])) OR (ResNet[Title/Abstract])) OR (GoogLeNet[Title/Abstract])) OR (Support vector machine[Title/Abstract])) OR (SVM[Title/Abstract])) OR (Gradient Boosting Machine[Title/Abstract])) OR (Nomogram[Title/Abstract])) OR (XGBoost[Title/Abstract])) OR (Adaboost[Title/Abstract])) OR (Decision tree[Title/Abstract])) OR (Naive Bayesian[Title/Abstract])) OR (Multilayer perceptron[Title/Abstract])) OR (Bayesian network[Title/Abstract])) OR (Prediction model[Title/Abstract])) OR (Risk model[Title/Abstract]))) | 463 |

**2.Cochrane**

| Search number | Query | Results |
| --- | --- | --- |
| #1 | MeSH descriptor: [Myopia] explode all trees | 1543 |
| #2 | (Myopia):ti,ab,kw OR (Myopias):ti,ab,kw OR (Nearsightedness):ti,ab,kw OR (Nearsightednesses):ti,ab,kw OR (myopy):ti,ab,kw | 3388 |
| #3 | (near-sightedness):ti,ab,kw OR (short-sightedness):ti,ab,kw OR (Shortsightedness):ti,ab,kw OR (Refractive errors):ti,ab,kw OR (Refractive error):ti,ab,kw | 1986 |
| #4 | (Short sight):ti,ab,kw OR (Near sight):ti,ab,kw OR (Myopic):ti,ab,kw | 1700 |
| #5 | #1 or #2 or #3 or #4 | 4853 |
| #6 | MeSH descriptor: [Machine Learning] explode all trees | 920 |
| #7 | (machine learning):ti,ab,kw OR (Transfer Learning):ti,ab,kw OR (Deep learning):ti,ab,kw OR (Ensemble Learning):ti,ab,kw OR (artificial intelligence):ti,ab,kw | 6428 |
| #8 | (random forest):ti,ab,kw OR (neural network):ti,ab,kw OR (neural networks):ti,ab,kw OR (K-Nearest Neighbor):ti,ab,kw OR (CNN):ti,ab,kw | 4171 |
| #9 | (AlexNet):ti,ab,kw OR (VGGNet):ti,ab,kw OR (ResNet):ti,ab,kw OR (GoogLeNet):ti,ab,kw OR (Support vector machine):ti,ab,kw | 557 |
| #10 | (SVM):ti,ab,kw OR (Gradient Boosting Machine):ti,ab,kw OR (Nomogram):ti,ab,kw OR (XGBoost):ti,ab,kw OR (Adaboost):ti,ab,kw | 1983 |
| #11 | (Decision tree):ti,ab,kw OR (Naive Bayesian):ti,ab,kw OR (Multilayer perceptron):ti,ab,kw OR (Bayesian network):ti,ab,kw OR (Prediction model):ti,ab,kw | 6942 |
| #12 | (Risk model):ti,ab,kw | 30138 |
| #13 | #6 or #7 or #8 or #9 or #10 or #11 or #12 | 42753 |
| #14 | #5 and #13 | 98 |

**3.Embase**

| Search number | Query | Results |
| --- | --- | --- |
| #1 | 'myopia'/exp | 32068 |
| #2 | myopia:ab,ti OR myopias:ab,ti OR nearsightedness:ab,ti OR nearsightednesses:ab,ti OR myopy:ab,ti OR 'near sightedness':ab,ti OR 'short sightedness':ab,ti OR shortsightedness:ab,ti OR 'refractive errors':ab,ti OR 'refractive error':ab,ti OR 'short sight':ab,ti OR 'near sight':ab,ti OR myopic:ab,ti | 40610 |
| #3 | #1 OR #2 | 48375 |
| #4 | 'machine learning'/exp | 412674 |
| #5 | 'machine learning':ab,ti OR 'transfer learning':ab,ti OR 'deep learning':ab,ti OR 'ensemble learning':ab,ti OR 'artificial intelligence':ab,ti OR 'random forest':ab,ti OR 'neural network':ab,ti OR 'neural networks':ab,ti OR 'k-nearest neighbor':ab,ti OR cnn:ab,ti OR alexnet:ab,ti OR vggnet:ab,ti OR resnet:ab,ti OR googlenet:ab,ti OR 'support vector machine':ab,ti OR svm:ab,ti OR 'gradient boosting machine':ab,ti OR nomogram:ab,ti OR xgboost:ab,ti OR adaboost:ab,ti OR 'decision tree':ab,ti OR 'naive bayesian':ab,ti OR 'multilayer perceptron':ab,ti OR 'bayesian network':ab,ti OR 'prediction model':ab,ti OR 'risk model':ab,ti | 344061 |
| #6 | #4 OR #5 | 556041 |
| #7 | #3 AND #6 | 633 |

**4.Web of science**

| Search number | Query | Results |
| --- | --- | --- |
| #1 | Myopia (Topic) OR Myopias (Topic) OR Nearsightedness (Topic) OR Nearsightednesses (Topic) OR myopy (Topic) OR near-sightedness (Topic) OR short-sightedness (Topic) OR Shortsightedness (Topic) OR Refractive errors (Topic) OR Refractive error (Topic) OR Short sight (Topic) OR Myopic (Topic) | 49012 |
| #2 | machine learning (Topic) OR Transfer Learning (Topic) OR Deep learning (Topic) OR Ensemble Learning (Topic) OR artificial intelligence (Topic) OR random forest (Topic) OR neural network (Topic) OR neural networks (Topic) OR K-Nearest Neighbor (Topic) OR CNN (Topic) OR AlexNet (Topic) OR VGGNet (Topic) OR ResNet (Topic) OR GoogLeNet (Topic) OR Support vector machine (Topic) OR SVM (Topic) OR Gradient Boosting Machine (Topic) OR Nomogram (Topic) OR XGBoost (Topic) OR Adaboost (Topic) OR Decision tree (Topic) OR Naive Bayesian (Topic) OR Multilayer perceptron (Topic) OR Bayesian network (Topic) OR Prediction model (Topic) OR Risk model (Topic) | 2927703 |
| #3 | #2 AND #1 | 3020 |
