# Supplementary material for: Machine Learning Approaches in High Myopia: Systematic Review and Meta-Analysis
Source: J Med Internet Res. 2025 Jan 3;27:e57644. doi: 10.2196/57644 (PMC11748443; doi:10.2196/57644)
Supplement: Multimedia Appendix 3 [file jmir_v27i1e57644_app3.docx]

**
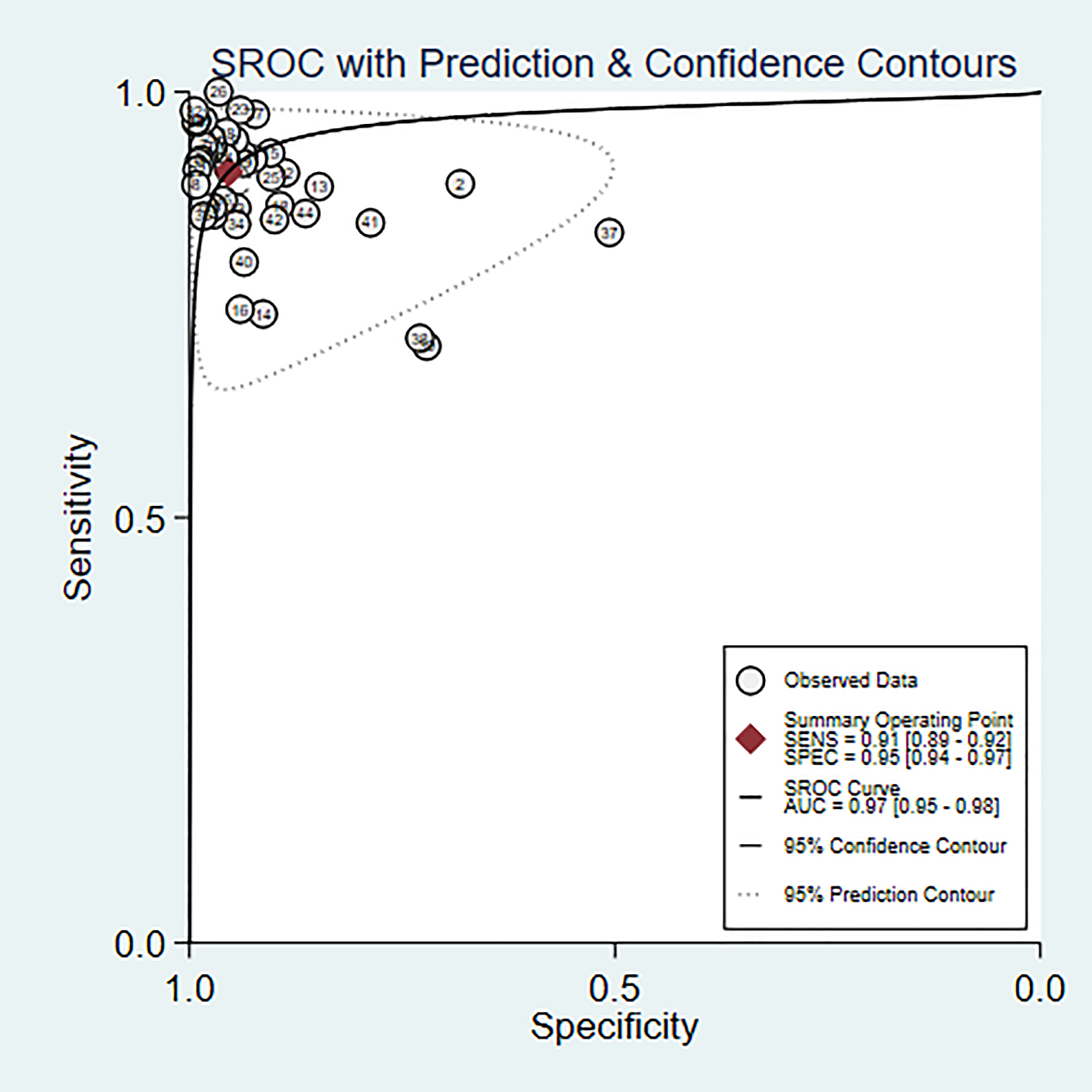
**

**Figure S1**. Summary receiver operating characteristic (SROC) curve for the meta-analysis of sensitivity and specificity of machine learning in detecting pathological myopia. (Note: 44 models from 20 machine learning studies on the diagnosis of pathological myopia; pooled SROC: 0.97 (95% CI: 0.95-0.98); SENS: sensitivity; SPEC: specificity)


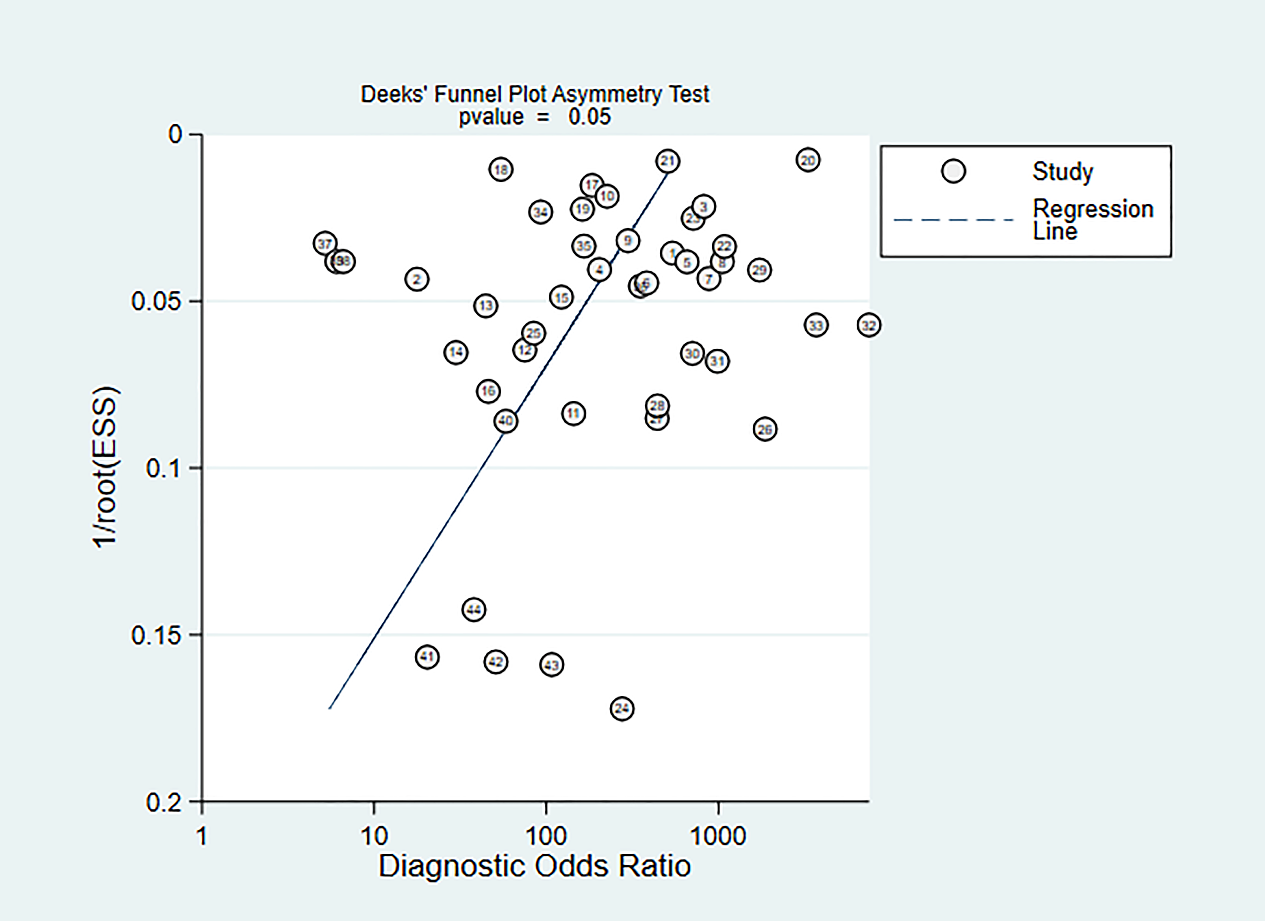


**Figure S2.** Funnel plot for the meta-analysis of sensitivity and specificity of machine learning in detecting pathological myopia. (Note: Deek's funnel plot indicated no substantial evidence of publication bias in the included machine learning models)


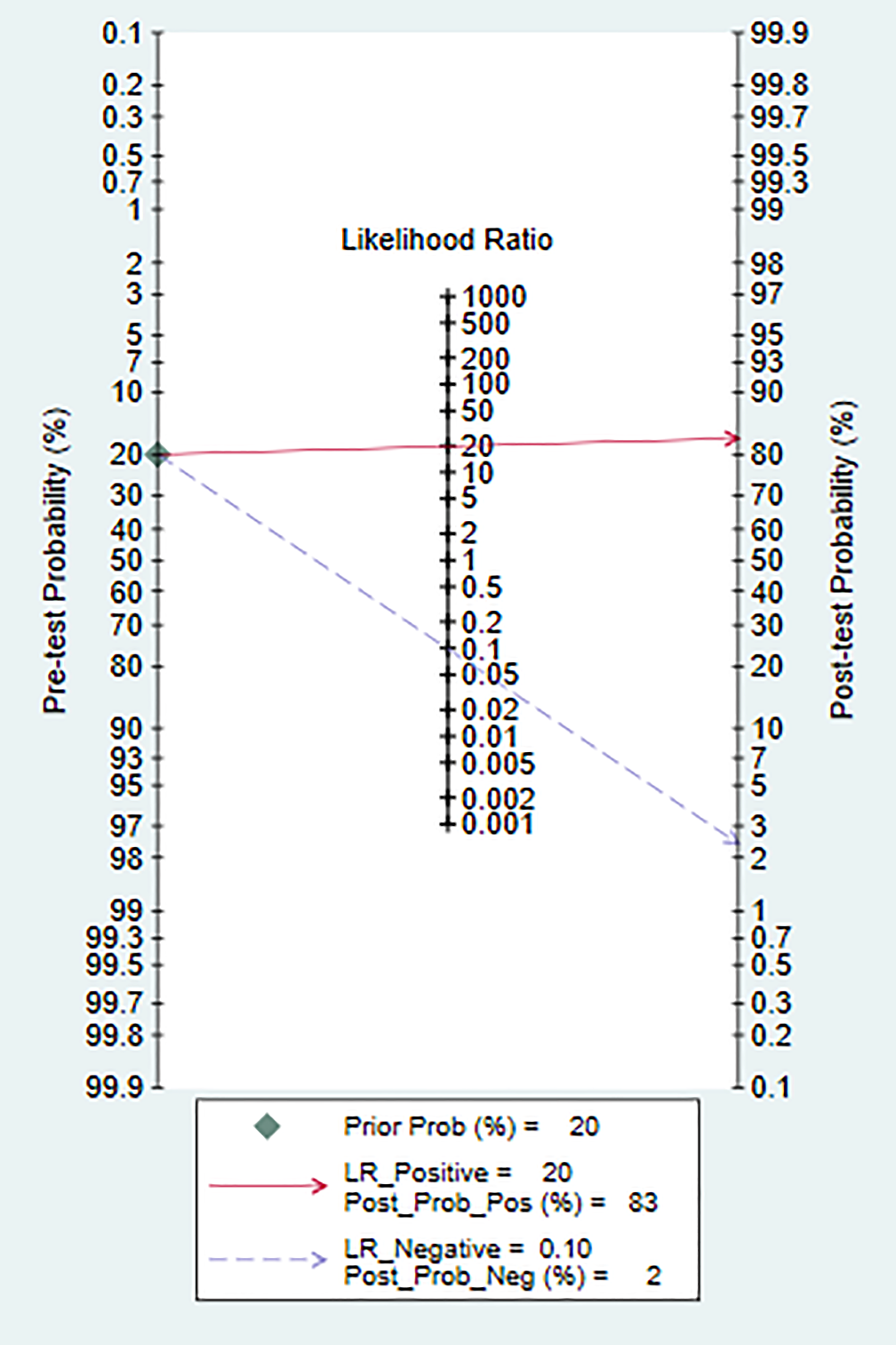


**Figure S3.** Column line graph for the meta-analysis of sensitivity and specificity of machine learning in detecting pathological myopia. (Note: Assuming that the prior probability of pathological myopia was 20%, if the result of machine learning was pathological myopia, then the probability of true pathological myopia would be 83%. If the result of machine learning was non-pathological myopia, then the probability of true pathological myopia would be 2% (i.e., the probability of true non-pathological myopia was 98%) )


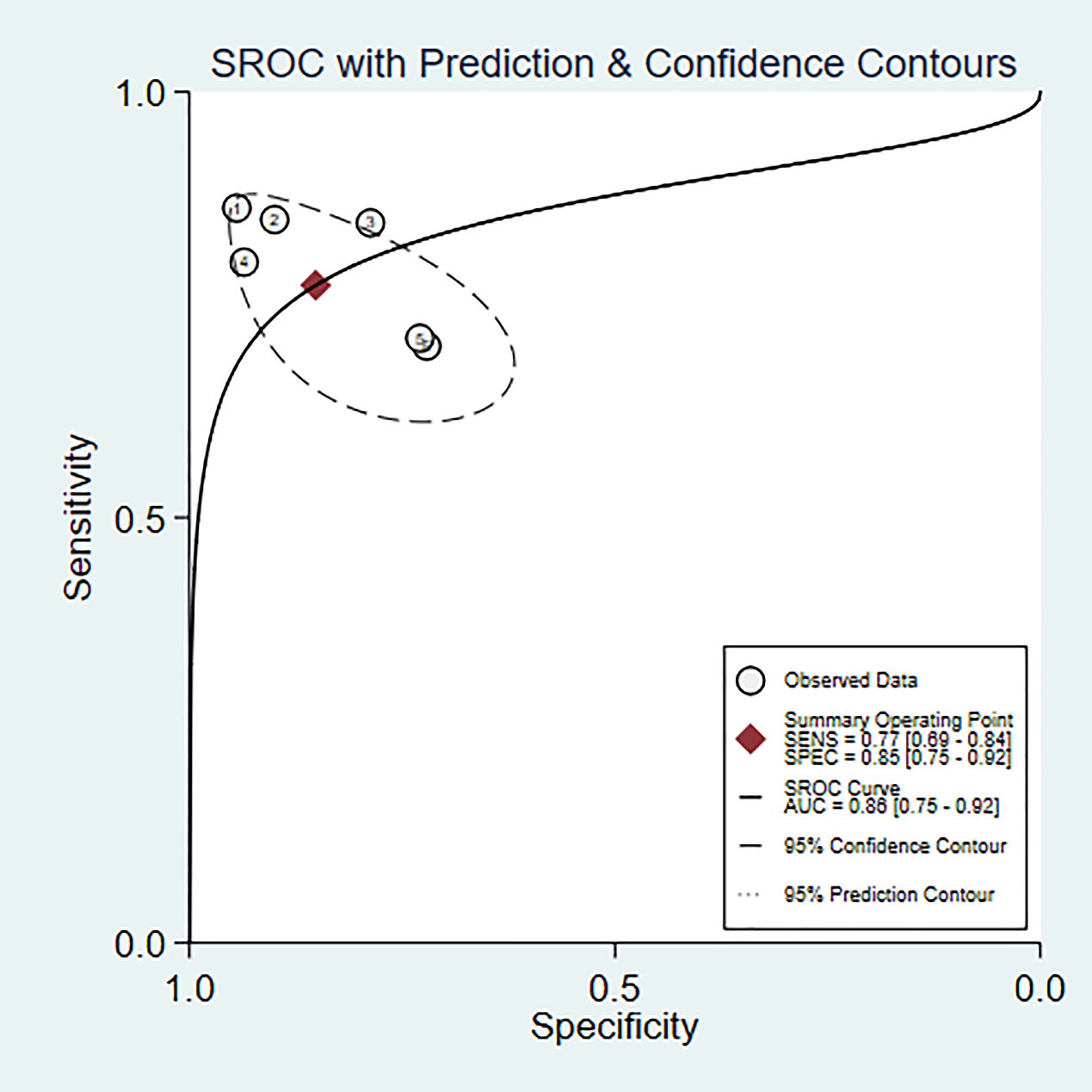


**Figure S4.** SROC curve for the meta-analysis of sensitivity and specificity of conventional machine learning (non-deep learning) in detecting pathological myopia. (Note: 6 models from 5 conventional machine learning (non-deep learning) studies on the diagnosis of pathological myopia; pooled SROC: 0.86 (95% CI: 0.75-0.92); SENS: sensitivity; SPEC: specificity)


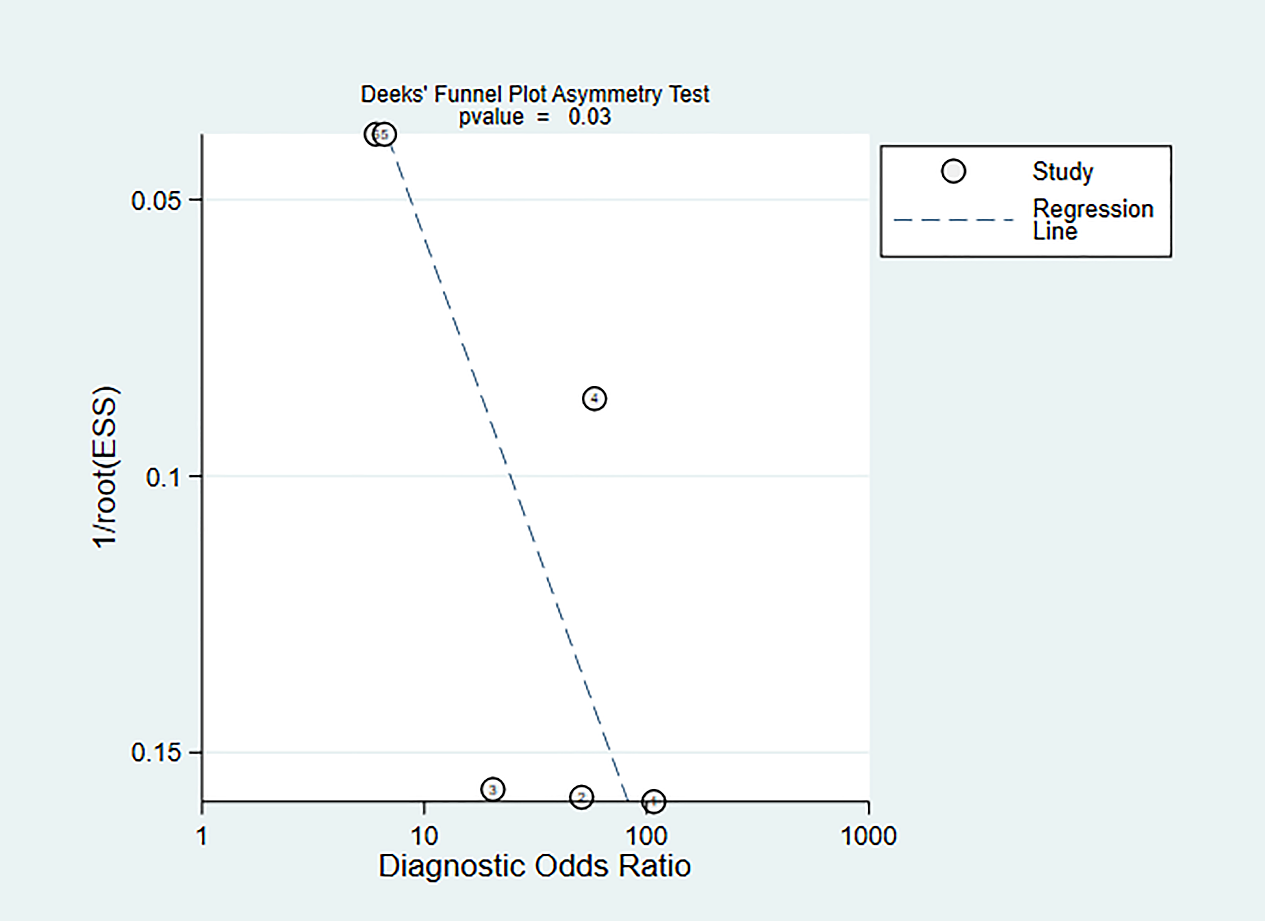


**Figure S5.** Funnel plot for the meta-analysis of sensitivity and specificity of conventional machine learning (non-deep learning) in detecting pathological myopia. (Note: Deek's funnel plot indicated the presence of publication bias in the conventional machine learning (non-deep learning) models.)


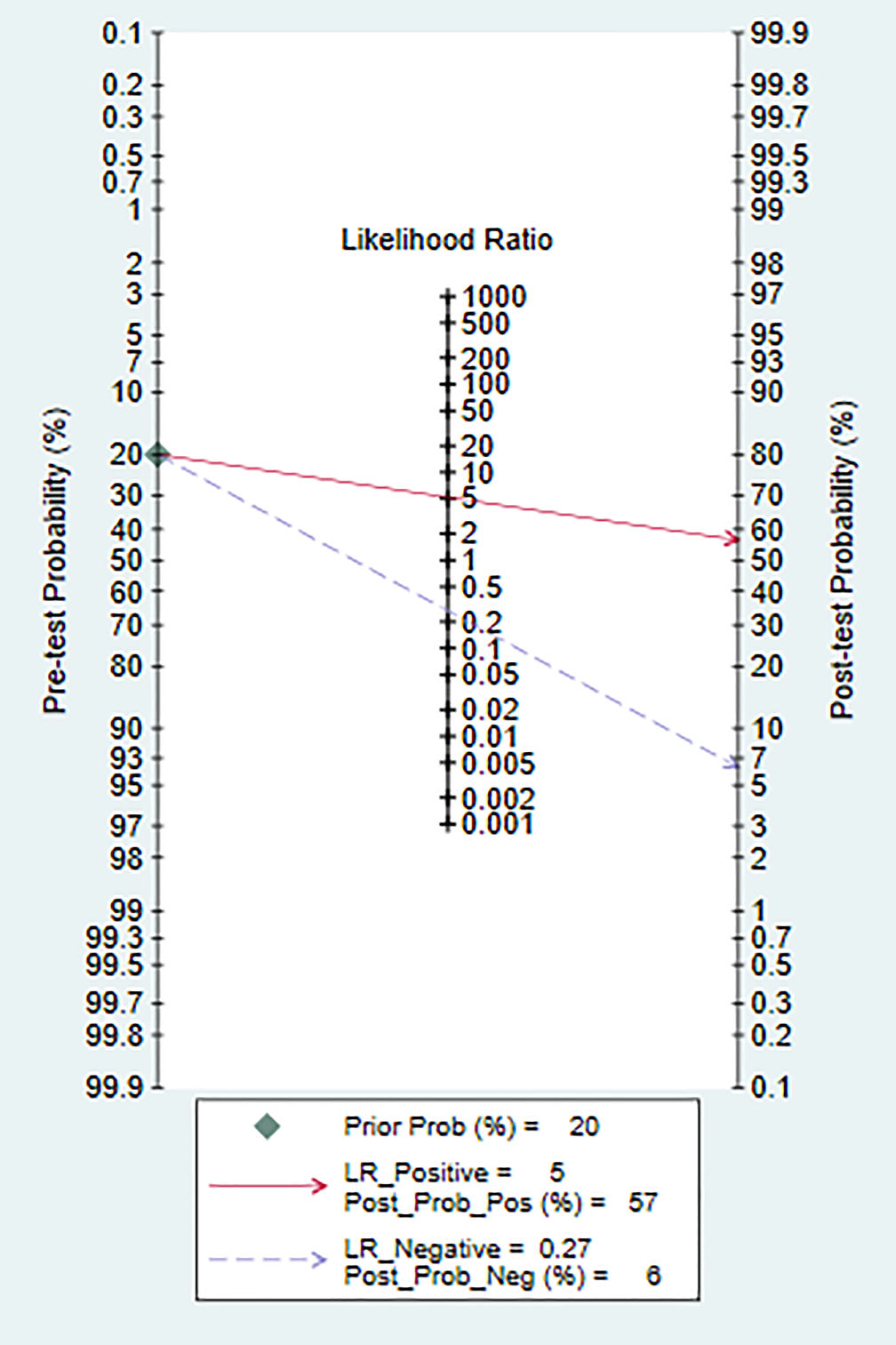


**Figure S6.** Column line graph for the meta-analysis of sensitivity and specificity of conventional machine learning (non-deep learning) in detecting pathological myopia. (Note: Assuming that the prior probability of pathological myopia for conventional machine learning (non-deep learning) was 20%, if the result of conventional machine learning (non-deep learning) was pathological myopia, then the probability of true pathological myopia would be 57%. If the result of conventional machine learning (non-deep learning) was non-pathological myopia, then the probability of true pathological myopia would be 6% (i.e., the probability of true non-pathological myopia was 94%))


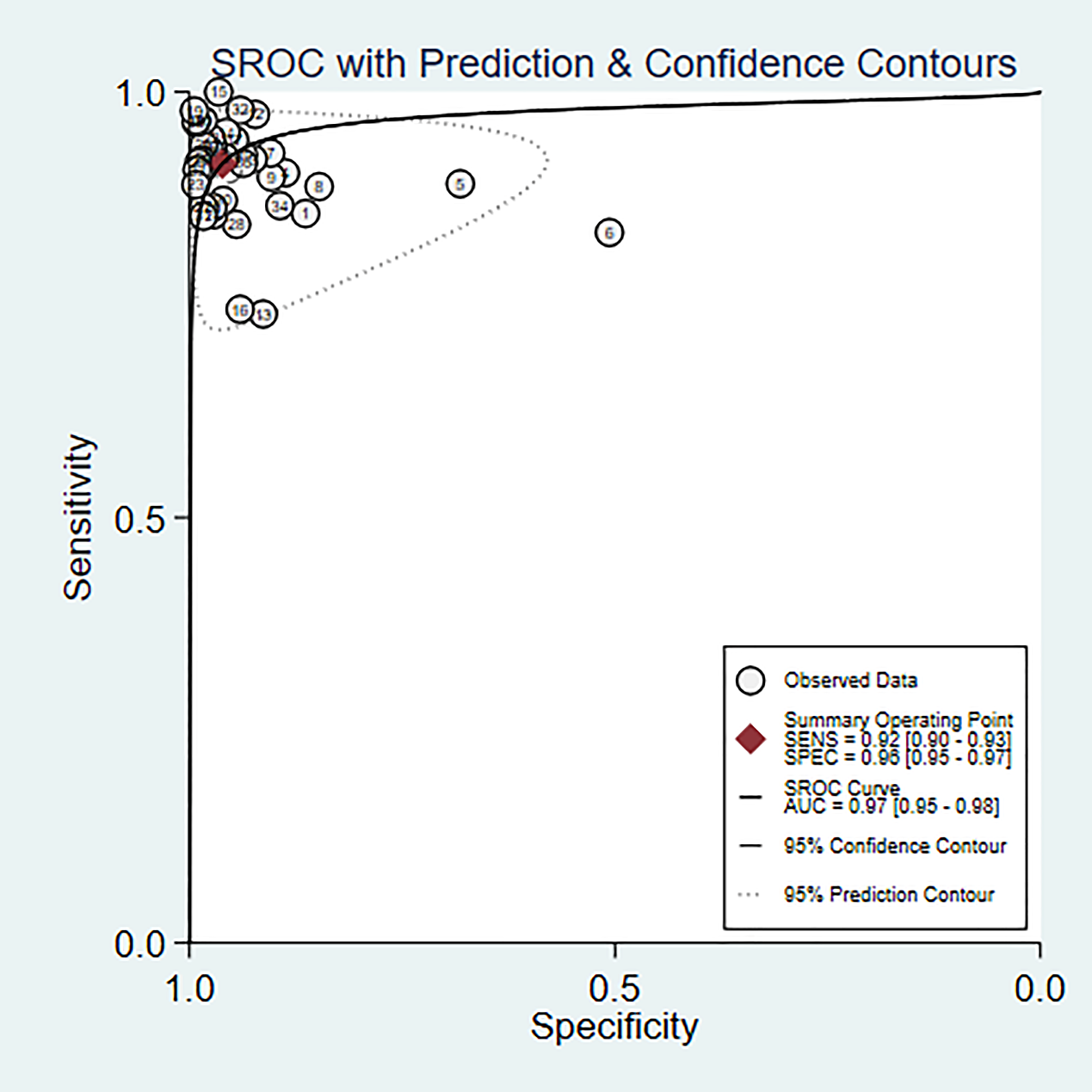


**Figure S7.** SROC curve for the meta-analysis of sensitivity and specificity of deep learning in detecting pathological myopia. (Note: 38 models from 15 deep learning studies on the diagnosis of pathological myopia; pooled SROC: 0.97 (95% CI: 0.95-0.98); SENS: sensitivity; SPEC: specificity)


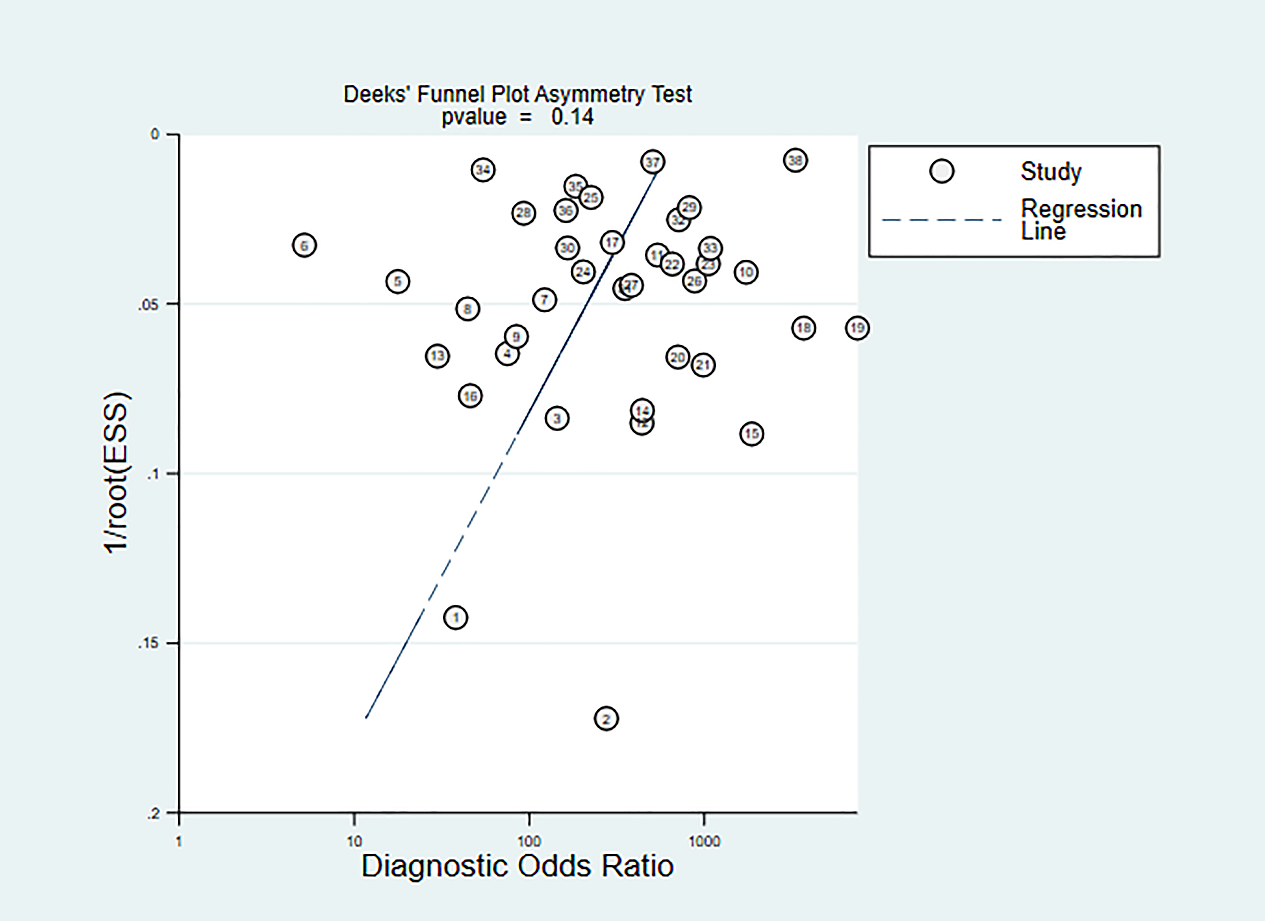


**Figure S8.** Funnel plot for the meta-analysis of sensitivity and specificity of deep learning in detecting pathological myopia. (Note: Deek's funnel plot revealed no remarkable publication bias in the deep learning models.)


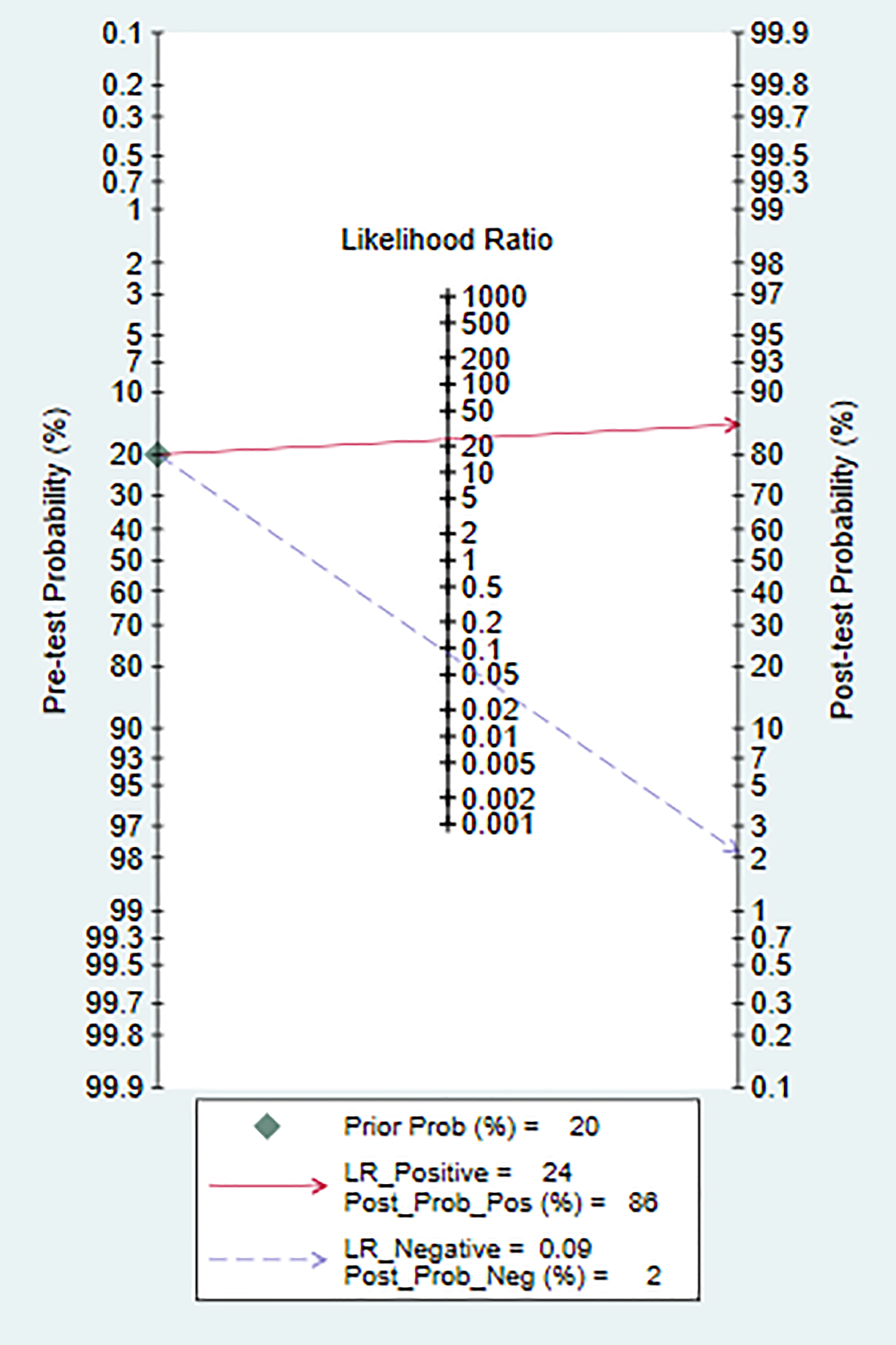


**Figure S9.** Column line graph for the meta-analysis of sensitivity and specificity of deep learning in detecting pathological myopia. (Note: Assuming that the prior probability of pathological myopia for deep learning was 20%, if the result of deep learning was pathological myopia, then the probability of true pathological myopia would be 86%. If the result of deep learning was non-pathological myopia, then the probability of true pathological myopia would be 2% (i.e., the probability of true non-pathological myopia was 98%))


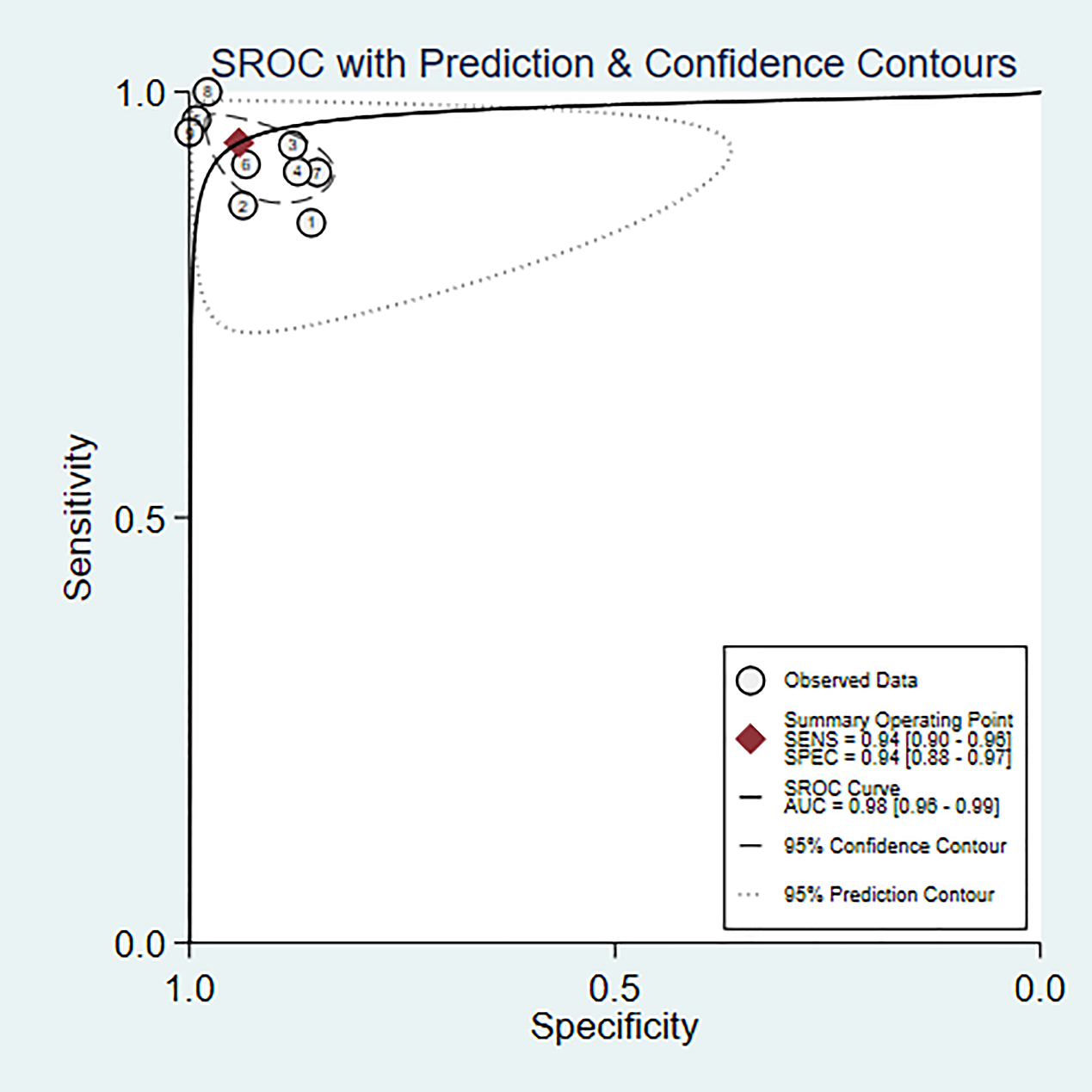


**Figure S10**. SROC curve for the meta-analysis of sensitivity and specificity of machine learning in detecting high myopia. (Note: 9 models from 6 machine learning studies on the diagnosis and prediction of high myopia; pooled SROC: 0.98 (95% CI: 0.96-0.99); SENS: sensitivity; SPEC: specificity)


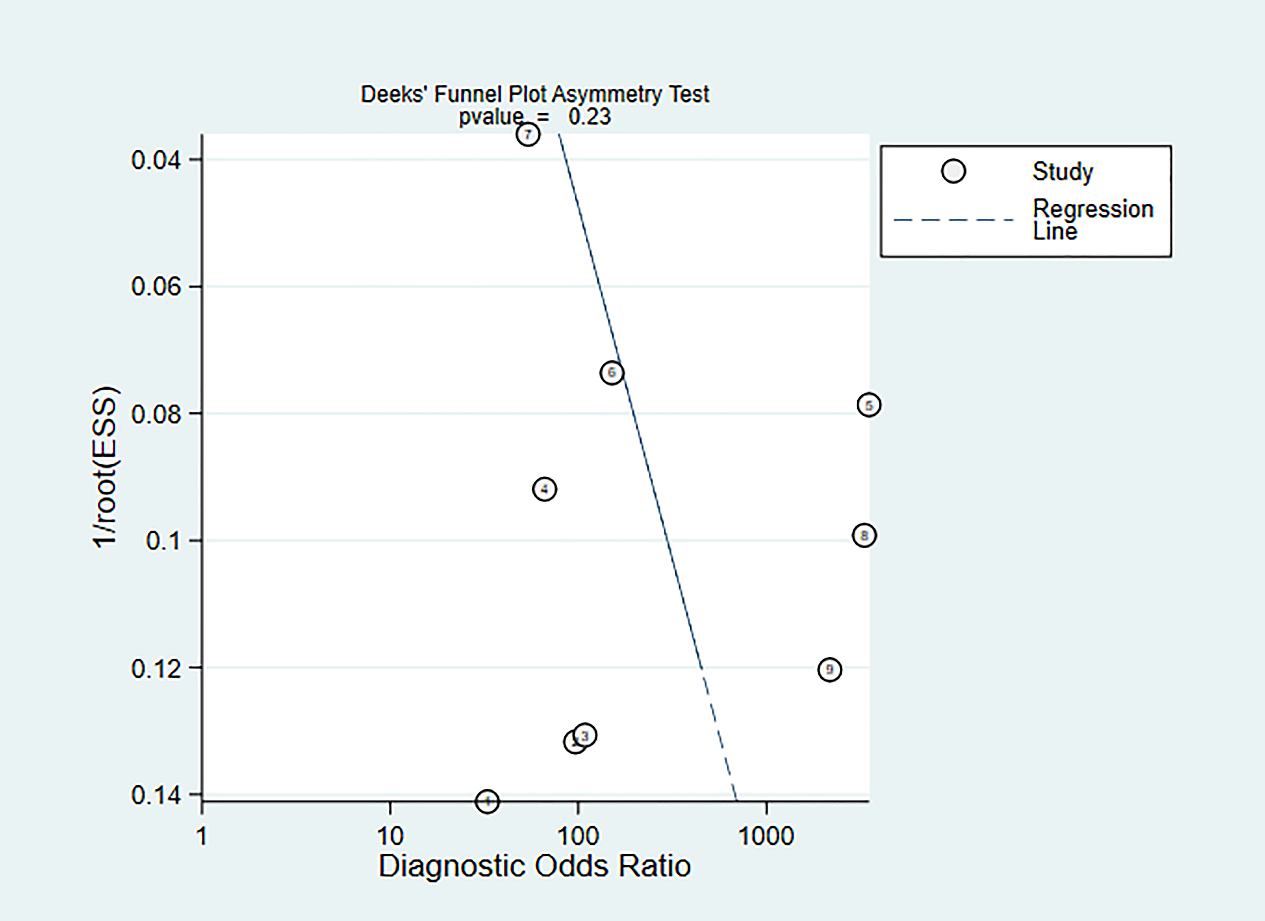


**Figure S11.** Funnel plot for the meta-analysis of sensitivity and specificity of machine learning in detecting high myopia. (Note: Deek's funnel plot indicated no substantial evidence of publication bias in the included machine learning models.)


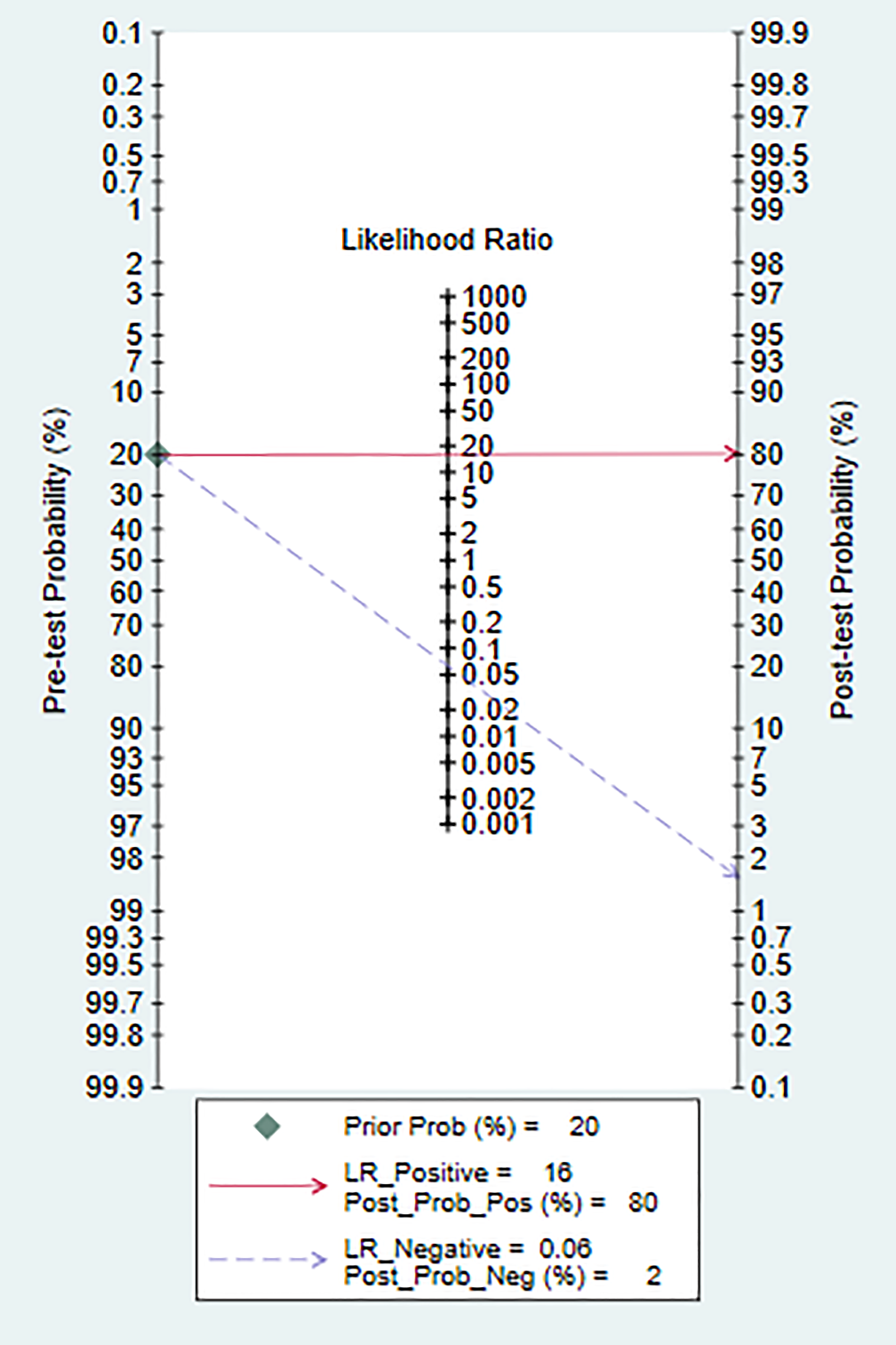


**Figure S12.** Column line graph for the meta-analysis of sensitivity and specificity of machine learning in detecting high myopia. (Note: Assuming that the prior probability of high myopia for machine learning was 20%, if the result of machine learning was high myopia, then the probability of true high myopia would be 80%. If the result of machine learning was non-high myopia, then the probability of true high myopia would be 2% (i.e., the probability of true non-high myopia was 98%))


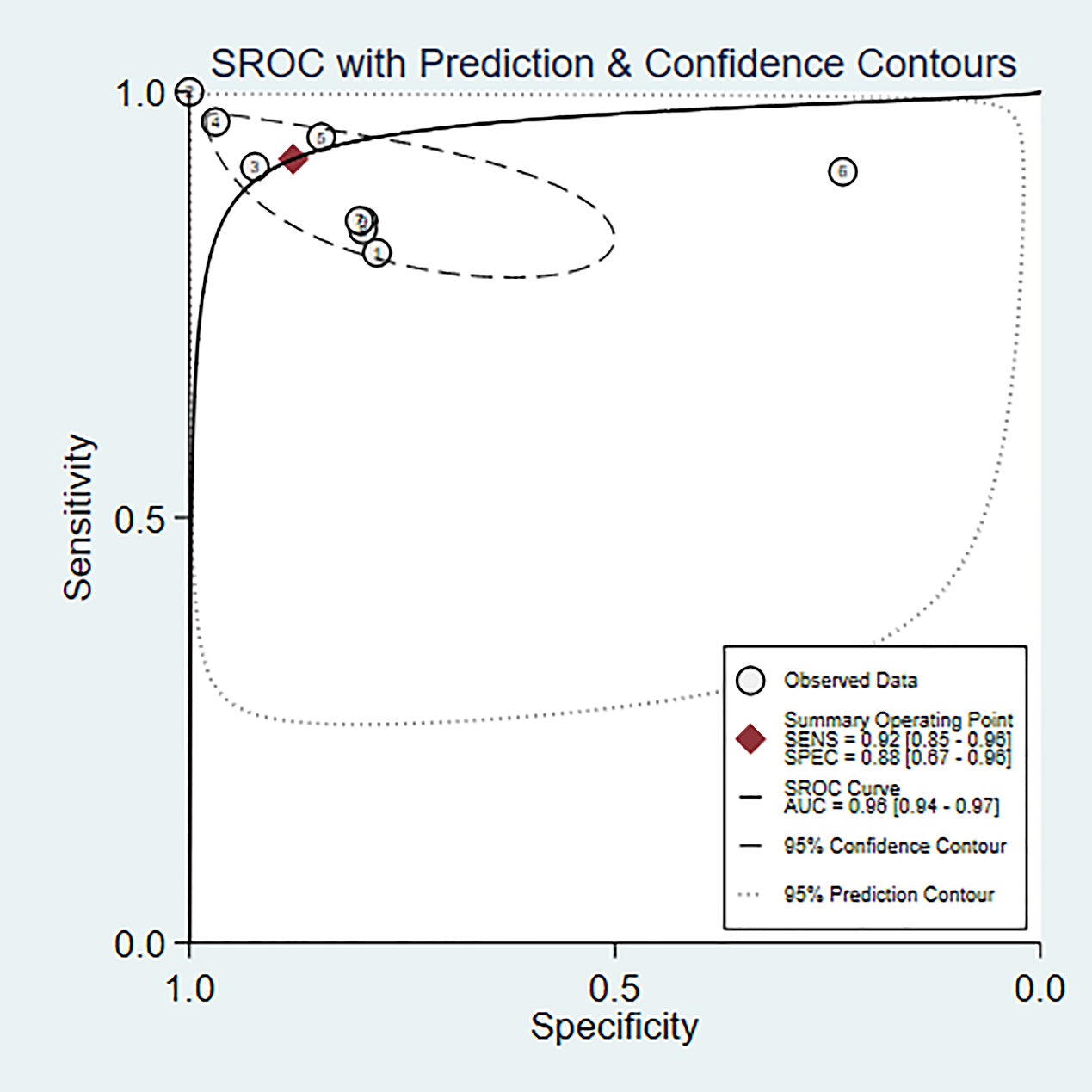


**Figure S13.** SROC curve for the meta-analysis of sensitivity and specificity of machine learning in detecting high myopia-associated glaucoma. (Note: 9 models from 6 machine learning studies on the diagnosis of high myopia-associated glaucoma; pooled SROC of 0.96 (95% CI: 0.94-0.97); SENS: sensitivity; SPEC: specificity)


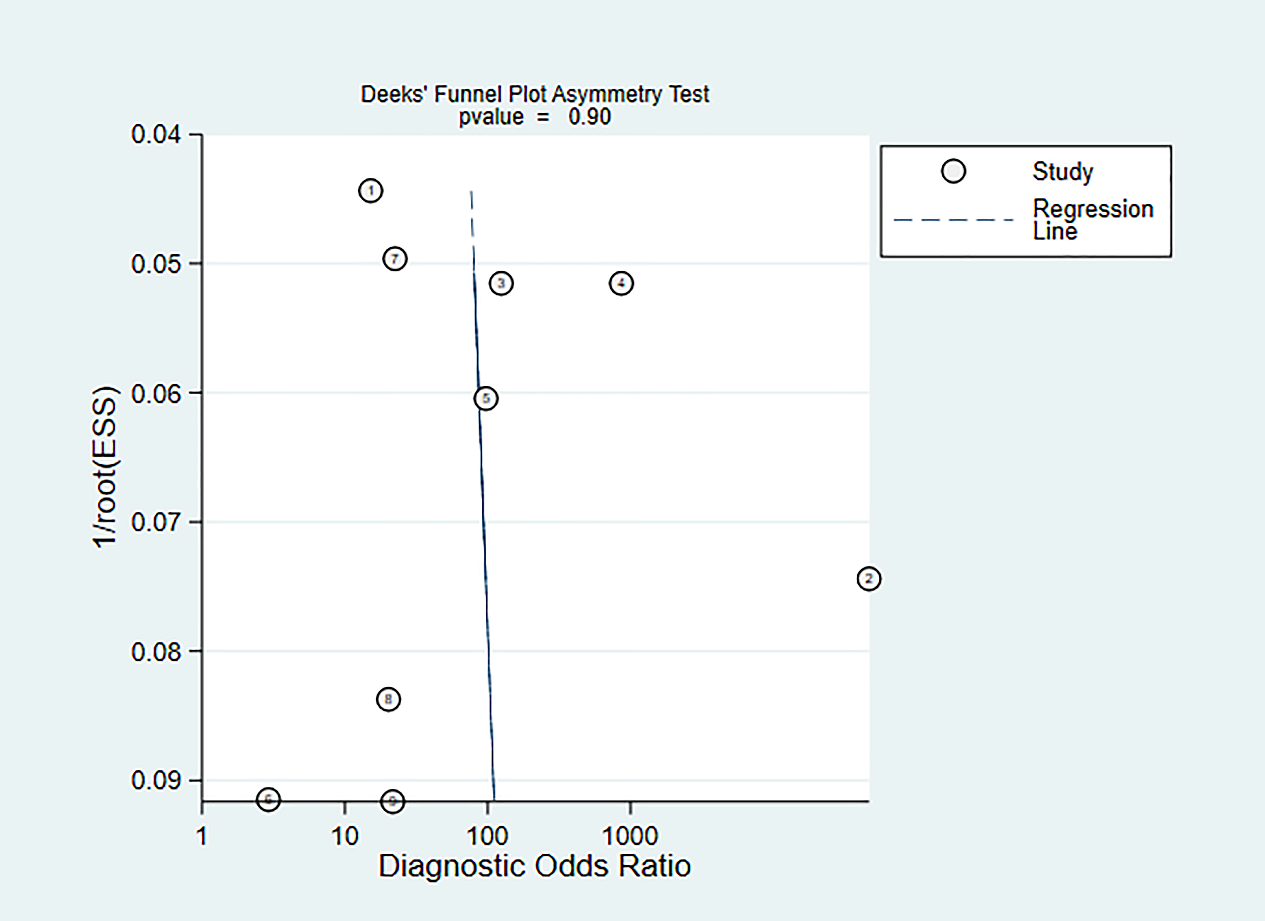


**Figure S14.** Funnel plot for the meta-analysis of sensitivity and specificity of machine learning in detecting high myopia-associated glaucoma. (Note: Deek's funnel plot indicated no substantial evidence of publication bias in the included machine learning models.)


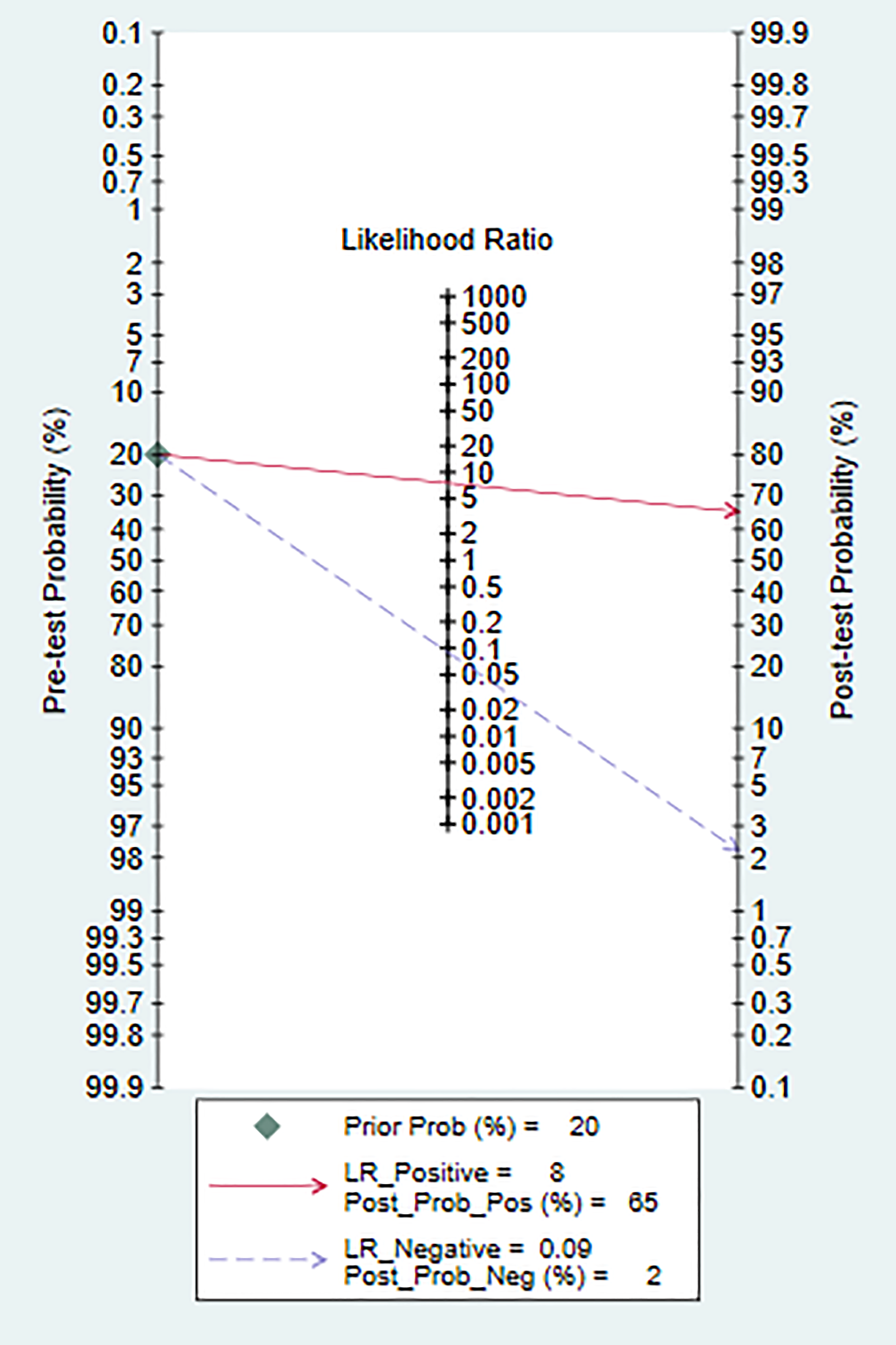


**Figure S15.** Column line graph for the meta-analysis of sensitivity and specificity of machine learning in detecting high myopia-associated glaucoma. (Note: Assuming that the prior probability of high myopia-associated glaucoma was 20%, if the result of machine learning was high myopia-associated glaucoma, then the probability of true high myopia-associated glaucoma would be 65%. If the result of machine learning was non-high myopia-associated glaucoma, then the probability of true high myopia-associated glaucoma would be 2% (i.e., the probability of true non-high myopia-associated glaucoma was 98%))
